# Assessing the reliability of cellular decision making from noisy, multidimensional single-cell TNF–NF-κB signaling data

**DOI:** 10.1371/journal.pcbi.1014608

**Published:** 2026-08-12

**Authors:** Ali Emadi, Tomasz Lipniacki, Andre Levchenko, Ali Abdi

**Affiliations:** 1 Arc Institute, Palo Alto, California, United States of America; 2 Institute of Fundamental Technological Research, Polish Academy of Sciences, Warsaw, Poland; 3 Yale Systems Biology Institute, Department of Biomedical Engineering, Yale University, New Haven, Connecticut, United States of America; 4 Center for Wireless Information Processing, Department of Electrical and Computer Engineering, New Jersey Institute of Technology, Newark, New Jersey, United States of America; 5 Department of Biological Sciences, New Jersey Institute of Technology, Newark, New Jersey, United States of America; University of California, UNITED STATES OF AMERICA

## Abstract

Cells make hard calls under noise. When signaling is abnormal, those calls can go wrong and drive pathological conditions and diseases. In this research, we develop a Neyman–Pearson (NP) detection-theory framework that maximizes probability of detection (*P*_D_) for a chosen false alarm probability (*P*_FA_), without requiring prior probabilities, using experimental single-cell measurements of NF-κB responses to tumor necrosis factor (TNF), a critical pathway involved in cell survival, apoptosis, immune signaling, and stress response, in wild-type and A20-deficient fibroblasts. We model log-responses as (multi)variate Gaussian and compute optimal thresholds, *P*_D_–*P*_FA_ trade-offs, and ROC curves at 30 minutes and 4 hours. The NP framework captures expected biology: *P*_D_ increases with TNF dose; wild-type cells outperform A20^-/-^ at matched conditions; and combining two time points (bivariate analysis) improves detection (e.g., for 0.0052 vs. 0.2 ng/mL, *P*_D_ rises from 0.71 (30 minutes) and 0.42 (4 hours) to 0.80 at *P*_FA_ = 0.1). The analysis recovers expected biology (higher TNF causes higher detectability; negative feedback lowers late responses) and flags cases where decision quality degrades (e.g., perturbations that blunt separation between conditions). The same recipe extends to multivariate readouts without changing the logic. A non-parametric (kernel-density) detector applied to the raw single-cell data reproduces these detection probabilities (|Δ*P*_D_| ≤ 0.092), confirming the conclusions do not rely on the Gaussian approximation. Overall, the NP detection framework provides a compact, quantitative score of pathway performance and failure. It turns noisy single-cell readouts into actionable decision metrics that compare doses, time points, and perturbations, and ultimately, can help explain when and how cellular decisions drift toward pathology.

## Author summary

Cells constantly sense their environment and make decisions based on chemical signals, but biological noise makes these decisions error-prone. When decision-making goes wrong, for instance due to mutations,diseases like chronic inflammation or cancer can result. In this study, we applied a well-established framework from signal detection theory, the Neyman-Pearson approach, to quantify how reliably cells can distinguish between different levels of the signaling molecule TNF. Using single-cell measurements from both normal cells and cells lacking the A20 feedback regulator, we computed detection and false alarm probabilities at different time points. We found that analyzing multiple time points together improves detection, that stronger signals are easier to detect, and that loss of A20 degrades decision quality—consistent with known biology. Our framework does not require assumptions about how likely each signal is, making it generalizable and broadly applicable. We believe this approach offers a practical, quantitative “scorecard” for cellular signaling performance that could help researchers understand when and why cellular decisions go wrong in disease.

## Introduction

**Fig 1:**
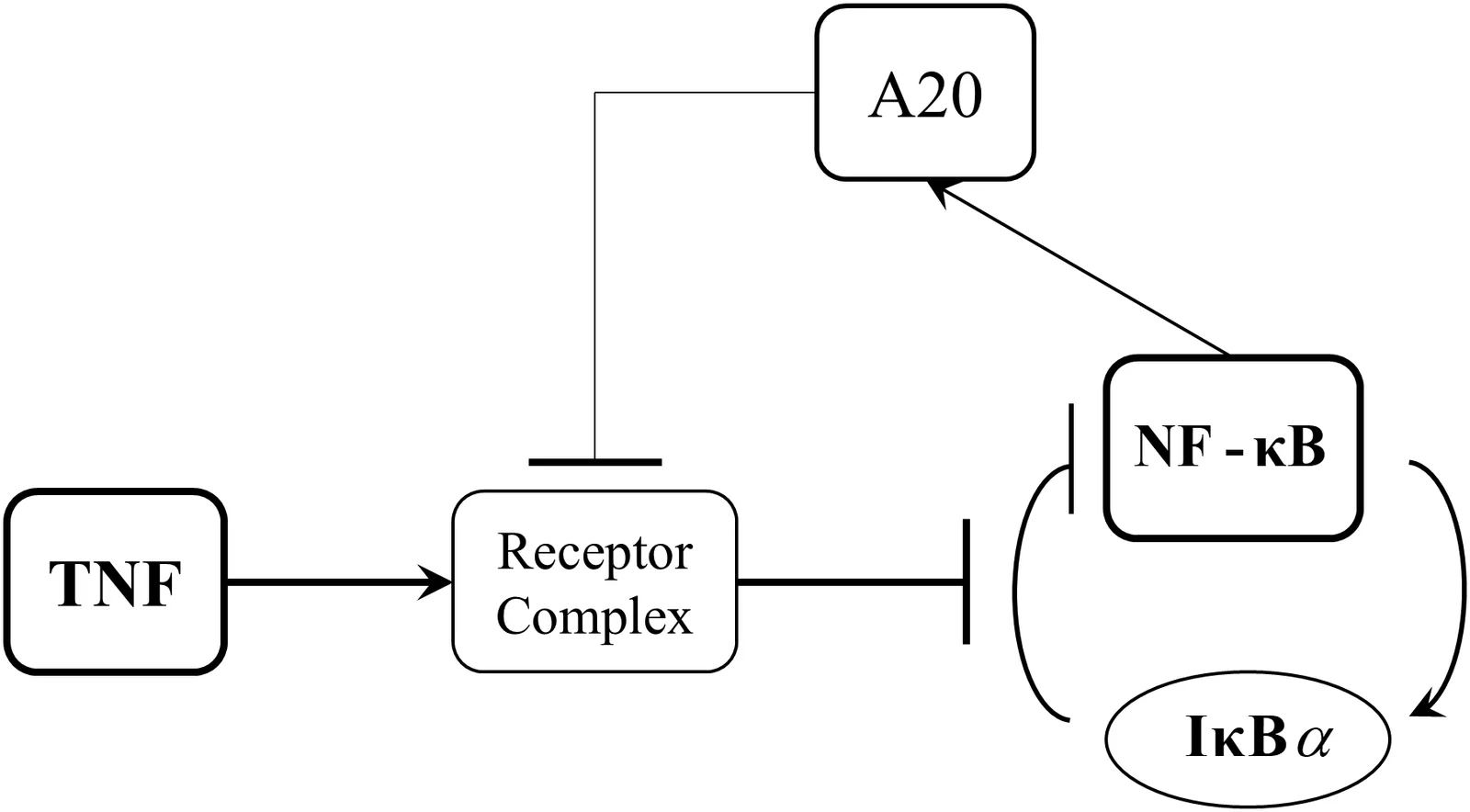
The Role of A20 in TNF–NF-κB signaling pathway.

In the field of cellular biology, the signal recognition and response of cells to environmental stimuli are key concepts. These processes are commonly involved in intricate biochemical signaling networks, where the strength and concentration of extracellular signals can influence cell fate and intercellular phenomena [1–3]. By comprehending the intricacies of such interactions, we can improve our understanding of cellular behavior, which may lead to novel approaches for the diagnosis and treatment of various pathological conditions. Therefore, it is crucial to study the mechanisms involved in this process to gain deeper insights into cellular decision-making. Molecular noise is a common phenomenon in biological systems [4], and its presence can impede the ability of cells to accurately sense and respond to various input signals [5]. The inherent stochasticity of biological noise renders cellular decision-making probabilistic in nature [5]. A wide range of potential outcomes can impact the cell’s behavior and the system’s overall function, each with varying degrees of likelihood. Even though biological noise presents significant challenges, researchers are actively seeking new ways to reduce its effects and improve the accuracy of cellular signaling and decision-making processes. Specifically, it has been shown that signal transduction noise arises from both extrinsic and intrinsic sources. Intrinsic noise is caused by the stochastic nature of biochemical reactions, which involve a small number of molecules. On the other hand, extrinsic noise results from variations in the levels of TNF receptors and cellular heterogeneity upon stimulation [6]. To gain a better understanding of the probabilistic nature of cellular decision-making, we employ a system model with explicit statistical parameters for alternative outcomes and quantify detection performance from single-cell data. Recently, similar models have used decision theory to derive optimal thresholds and error probabilities from such measurements. The objective of such a model is to assess the system’s capability to accurately detect the presence or absence of the desired signal. In those studies, the prior probabilities for signal presence/absence were assumed to be known, typically equal, that is 0.5/0.5, and then the maximum likelihood decision approach was applied [7–9]. It is important to consider a different modeling approach when the prior probabilities of signal presence are unknown. This paper introduces a signal detection model where the cell maximizes the probability of detecting the signal for any given false alarm probability. The signal detection model used in this study is based on the Neyman-Pearson signal detection theorem, which takes into account the probability of falsely detecting a signal when it is absent, also known as the false alarm probability. These quantitative and probabilistic models also have the potential to shed light on the stochastic signaling mechanisms and phenotype induction associated with genetic mutations that cause diseases [10]. By providing a deeper understanding of these mechanisms, our proposed model may contribute to the development of novel approaches for the diagnosis and treatment of genetic disorders. In order to validate such a method in a biologically meaningful context, we utilized a signaling pathway involving the regulation of the transcription factor Nuclear Factor κB (NF-κB) by tumor necrosis factor (TNF) (Fig 1) [5]. This allowed us to investigate the method’s effectiveness in a real-world scenario and assess its potential for future applications. TNF is a crucial antiviral cytokine that can cause significant damage to healthy host tissues. In addition to its role in activating NF-κB, a critical gene regulator involved in cell survival, viral replication, and pathological processes such as autoimmune diseases, various types of cancer, and inflammation, TNF has also been shown to mediate both anti-apoptotic and pro-apoptotic signals and can even trigger necroptosis, a type of pro-inflammatory cell death [11]. Upon activation, NF-κB can act to inhibit apoptosis in cells. The presence of the A20 molecule can also help reduce noise in the signaling pathway by acting as negative feedback to the information bottleneck. However, the effect of A20 activation on the amount of information attained in the system is variable and can either increase or decrease information [5]. The loss or mutation of the A20 molecule, inhibitory feedback of the NF-κB pathway, has been implicated in various pathological conditions such as cancer and chronic inflammation [12,13]. The negative feedback mediated by A20 can result in the reduction of NF-κB levels, leading to downstream effects on cellular signaling. Also, A20 deficiency may contribute to cancer development and lead to significant pathological conditions. This effect is believed to occur due to the persistent activation of anti-apoptosis genes mediated by NF-κB, as described in [14]. In response to NF-κB activation, the transcription of early genes, including cytokines, can be upregulated [7]. Furthermore, tumor microenvironments often contain heterogeneous cell populations with varying degrees of A20 expression, which makes mixed populations particularly relevant for understanding signal detection in cancer contexts.

The objective of this paper is to demonstrate the applicability of the proposed statistical decision-making framework to diverse molecular signaling systems. We aim to showcase the versatility and effectiveness of this framework across different biological contexts when there is no knowledge of priors. The aim is twofold: first, to observe how the suggested decision model is expanded to examine various time points in the process of signaling outcomes using statistical multi-dimensional analysis for the TNF–NF-κB. Second, using the widely recognized graphical tool, the receiver operating characteristic (ROC), to visualize cell decision-making performance in both normal and abnormal conditions. The overall objective is to determine the probabilities of cell decision-making and establish a correlation between statistical parameters and metrics with biological discoveries.

## Results

Based on single-cell data analysis of the TNF–NF-κB signaling pathway, it has been revealed that a cell can differentiate between low and high TNF level concentrations at the input [5]. The presence of molecular noise may impede the cell’s ability to accurately discriminate the strength of the input signals and external stimulations. Consequently, it affects the cellular decision-making results [5]. As previously noted, due to numerous diseases and pathological conditions that may arise from unanticipated and altered cellular decisions, there is a keen interest in devising suitable models for the characterization and quantification of molecular signal detection factors and cellular decision-making.

**Fig 2:**
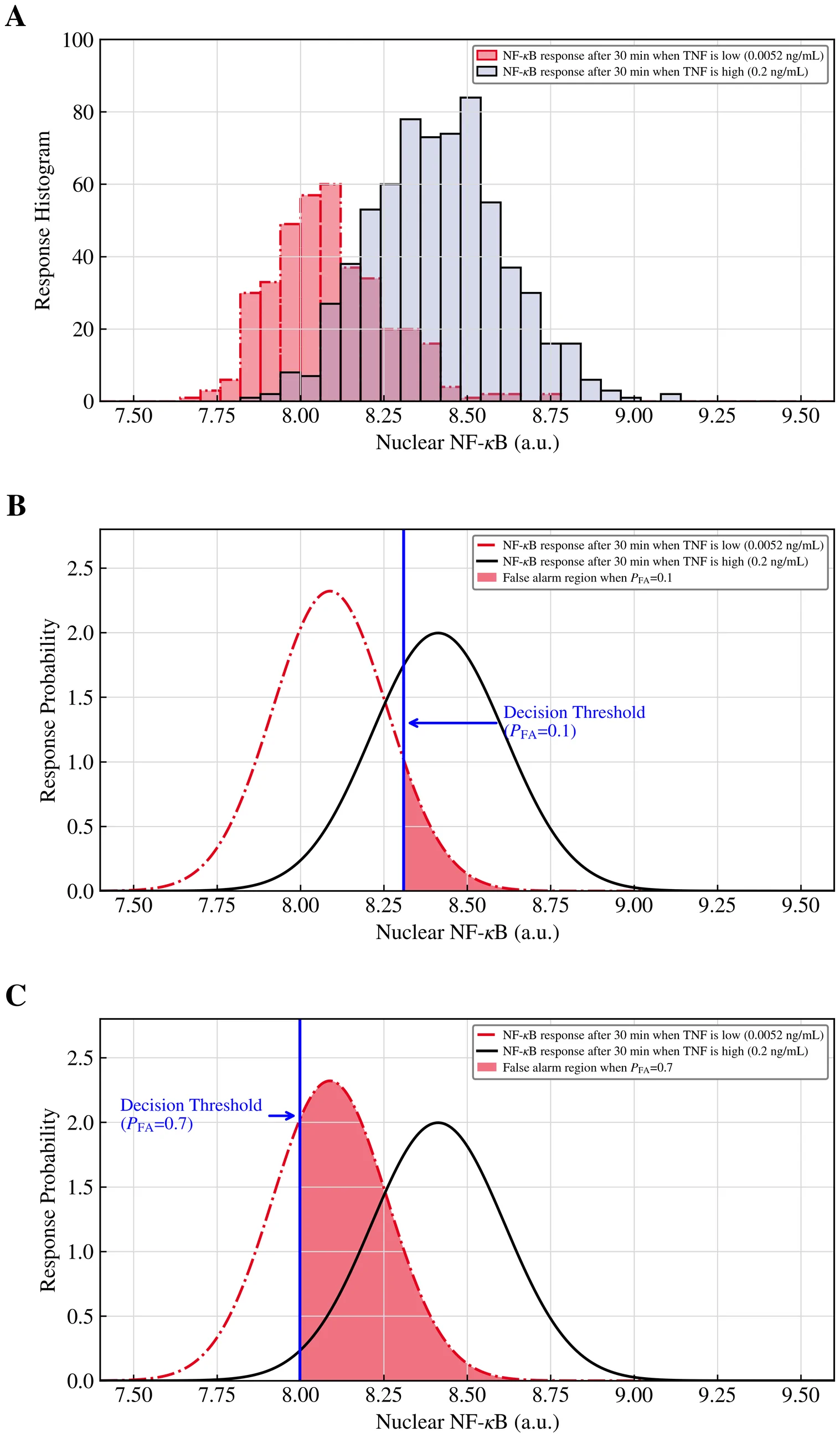
Univariate Neyman–Pearson detection of TNF level from single-cell NF-κB responses. **A)** Histograms of measured NF-κB concentrations for low TNF 0.0052 ng/mL vs. high TNF of 0.2 ng/mL. **B)** Gaussian PDF of NF-κB responses to low and high TNF levels after 30 minutes of exposure including the optimal decision threshold when *P*_FA_ = 0.1 shown as blue vertical line and the *P*_FA_ associated region. **C)** Same PDFs when *P*_FA_ = 0.7 and its corresponding region.

To analyze the proposed cellular decision-making framework for the signaling pathway in Fig 1, using nuclear NF-κB responses measured at the output upon receiving various TNF concentration levels at the system input, we focus on two types of decision probabilities as the desired metrics: probability of false alarm *P*_FA_, and probability of detection *P*_D_ (defined in METHODS). We evaluate the proposed model by choosing 0.0021, 0.0052, and 0.013 ng/mL as the low TNF levels (null hypothesis H_0_) vs. 0.2, 0.51, 1.3, and 50 ng/mL for high TNF levels (alternative hypothesis H_1_). For instance, Fig 2A shows the histograms of NF-κB responses of hundreds of cells after 30 minutes of exposure to a low TNF level of 0.0052 ng/mL vs. a high TNF level of 0.2 ng/mL. Fig 2B shows the associated univariate PDFs of the responses in Fig 2A, including the optimal decision threshold value when *P*_FA_ = 0.1 obtained by the Neyman-Pearson theorem (details in METHODS). The vertical blue line as the optimal decision threshold is the best value that maximizes the probability of detection *P*_D_, at *P*_FA_ = 0.1. Based on the definition in the Equation (4), the right-side area of the optimal decision threshold line, shaded in light red, represents the probability of false alarm *P*_FA_ region. Also, the probability of detection *P*_D_ region is on the right side of the obtained decision threshold under the associated PDF curve of high TNF level (hypothesis H_1_), which is not shaded due to possible overlap with the *P*_FA_ region. Fig 2C also illustrates the same PDFs for both low and high TNF levels in Fig 2B, but includes the shaded false alarm region when *P*_FA_ = 0.7. The expansion of the shaded *P*_FA_ region is expected. This increase in threshold enlarges the *P*_D_ region as well, which means a higher *P*_D_ value.

**Fig 3:**
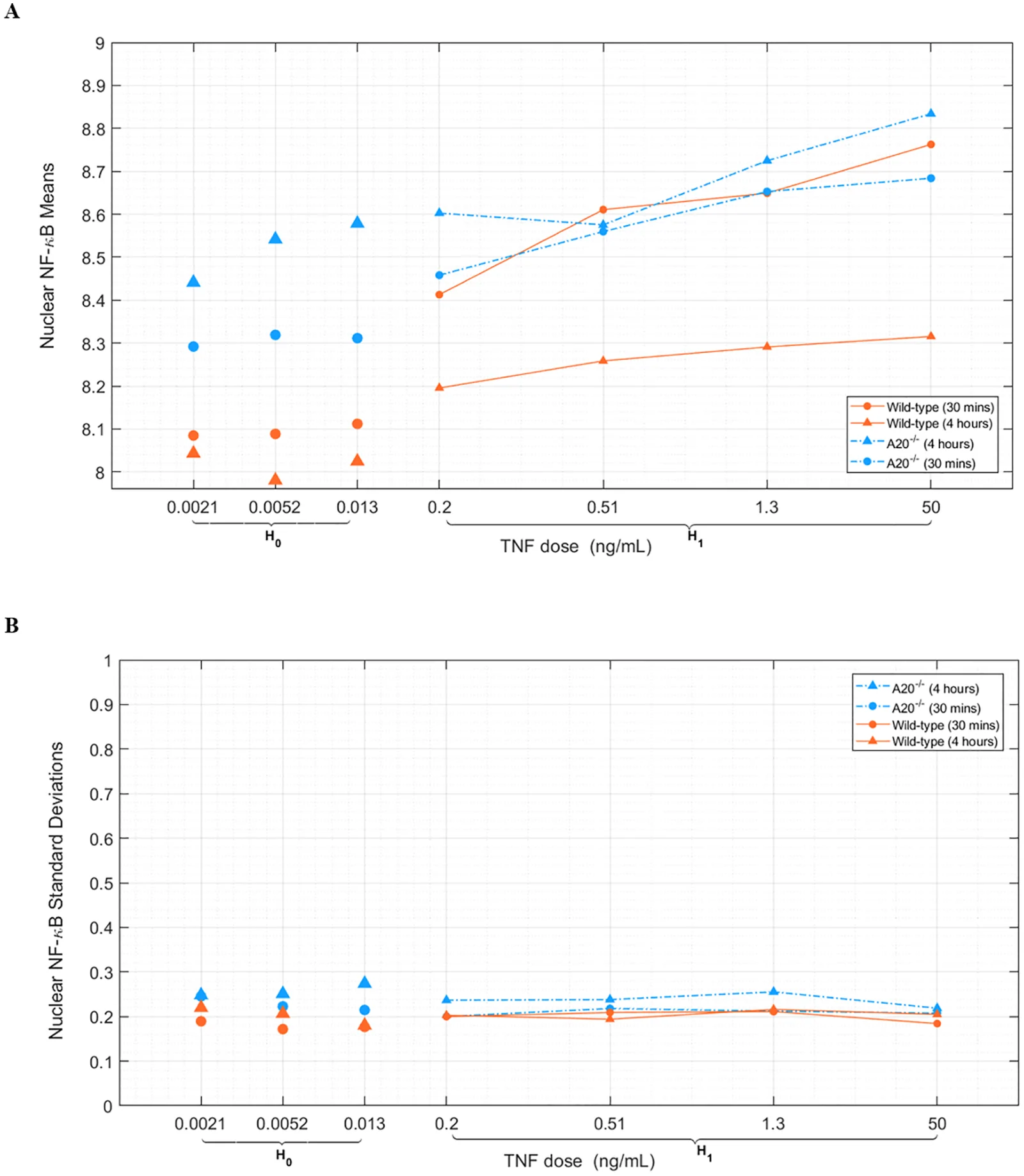
Dynamic range of responses to various TNF levels. **A)** Nuclear NF-κB means for various TNF levels. **B)** Standard deviations of Nuclear NF-κB in response to different TNF levels.

**Fig 4:**
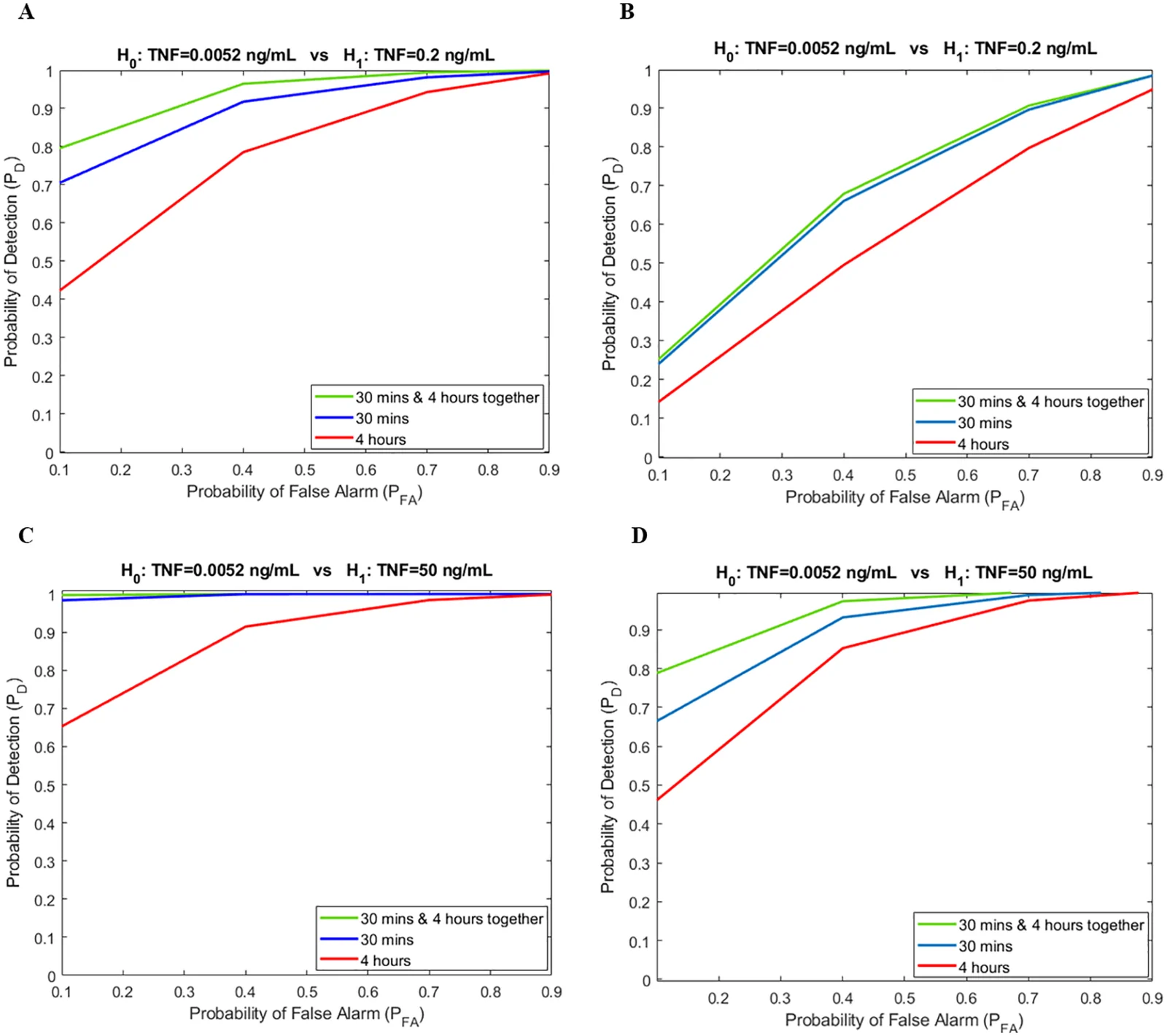
Receiver operating characteristic (ROC) curves for TNF–NF-κB signaling pathway evaluated using Neyman-Pearson theorem concepts when low TNF, H_0_ is 0.0052 ng/mL. **A)** Wild-type cells result for high TNF, H_1_ equal to 0.2 ng/mL. **B)** A20^−/−^ cells results for high TNF, H_1_ equal to 0.2 ng/mL. **C)** Wild-type cells result for high TNF, H_1_ equal to 50 ng/mL. **D)** A20^−/−^ cells results for high TNF, H_1_ equal to 50 ng/mL.

As a concise demonstration of the utilized experimental data, Fig 3A demonstrates the mean values of cells’ responses to different TNF levels in NF-κB after 30 minutes of TNF exposure. That is one of the important variables that can help to justify ROCs behaviors in Fig 4, as well. Also, this figure tells us that as the TNF level increases in each case, the corresponding mean increases as well due to the shifting of histograms to the right in the coordinate system. Fig 3B shows the standard deviation of cells’ responses to different TNF levels in NF-κB after 30 minutes of TNF exposure, where there are no high differences and fluctuations between various TNF levels’ standard deviations. When it comes to analyzing data, we invoke ROC (explained in METHODS) curves, here to see how the signaling system performs. Fig 4 shows the hypothesis testing problems of 0.0052 ng/mL as the low TNF level vs. 0.2 ng/mL and 50 ng/mL as the two high TNF levels over two different time points after TNF exposure, 30 minutes and 4 hours (individually and jointly analyzed) in both wild-type and A20^−/−^ cells. First, by comparing Fig 4C with Fig 4A associated with wild-type cells for different high TNF levels, 50 ng/mL and 0.2 ng/mL, respectively, this suggests that there is a visible reduction in all ROCs when the high TNF level decreases from 50 ng/mL to 0.2 ng/mL, which is consistent with our earlier analysis. A lower concentration of high TNF, like 0.2 ng/mL, has a closer histogram to the low TNF of 0.0052 ng/mL than the highest high TNF, which is 50 ng/mL.

Another important observation could be about the comparison of 4-hour ROCs with 30-minute ROCs for both high TNF levels, 0.2 ng/mL and 50 ng/mL, where we can say the 4-hour curves are below the 30-minute curves due to the activation of the negative feedback over time that reduces NF-κB level after 4 hours. In A20^−/−^ cells, we expect a shallower late decline than in wild-type, because the negative feedback that normally lowers the 4-hour response is largely absent; the A20^−/−^ 4-hour *P*_D_ nonetheless remains below its 30-minute value (Tables 1 and 2), since the H_0_–H_1_ separation is still smaller at 4 hours. The transition from wild-type to A20^−/−^ is like shifting the corresponding histograms to the right-hand side of the coordinate system. The means figure, Fig 3A, justifies this claim very well through a comparison of associated mean values of low and high TNF levels in the corresponding hypothesis testing. Both H_0_ and H_1_ histograms in A20^−/−^ have shifted to the right, and the means are good representatives of their histograms or their PDFs. In those figures, we can clearly see that the difference between the H_0_ means and H_1_ means plays an important role in the *P*_D_ of the Neyman-Pearson decision-making framework. Because, e.g., as the means become farther away, it seems like the histograms or PDFs are well-separated, and we can say the probability of detection is higher because the PDFs or histograms are nearly well separated. Also, these can be pointed out as the opposite, as well. Those cases with lower *P*_D,_ in each case, have a closer means of H_0_ and H_1_. As we can see, analyzing both time points, 30 minutes and 4 hours together, is like a bivariate PDF that can improve the decision-making performance. The bivariate case almost always stays either very close to the one with higher *P*_D_ or at the top of each individual 30-minute and 4-hour time points, reasonably. Moreover, by comparing the above ROCs from left (Fig 4A and 4C) to the right (Fig 4B and 4D), cell type transition from wild-type to A20^−/−^, we always notice a reduction in all cases, 30 minutes, 4 hours, and the bivariate scenario, as well.

**Table 1:** Decision Probabilities of wild-type and A20^−/−^ cells for TNF = 0.0052 ng/mL vs. TNF = 0.2 ng/mL.

Decision Probability	Cell type	30 minutes	4 hours	(30 minutes & 4 hours)
***P***_**D**_ **(at *P***_**FA**_ **= 0.1)**	WT	**0.71** (0.63-0.75)	**0.42** (0.29-0.50)	**0.8** (0.73-0.83)
	A20^−/−^	**0.24** (0.16-0.31)	**0.14** (0.11-0.21)	**0.26** (0.20-0.34)

**Table 2:** Decision Probabilities of wild-type and A20^−/−^ cells for TNF = 0.0052 ng/mL vs. TNF = 50 ng/mL.

Decision Probability	Cell type	30 minutes	4 hours	(30 minutes & 4 hours)
***P***_**D**_ **(at *P***_**FA**_ **= 0.1)**	WT	**~0.99** (0.99-1.00)	**0.65** (0.52-0.71)	**1.00** (1.00-1.00)
	A20^−/−^	**0.67** (0.54-0.74)	**0.46** (0.30-0.57)	**0.79** (0.72-0.85)

**Fig 5:**
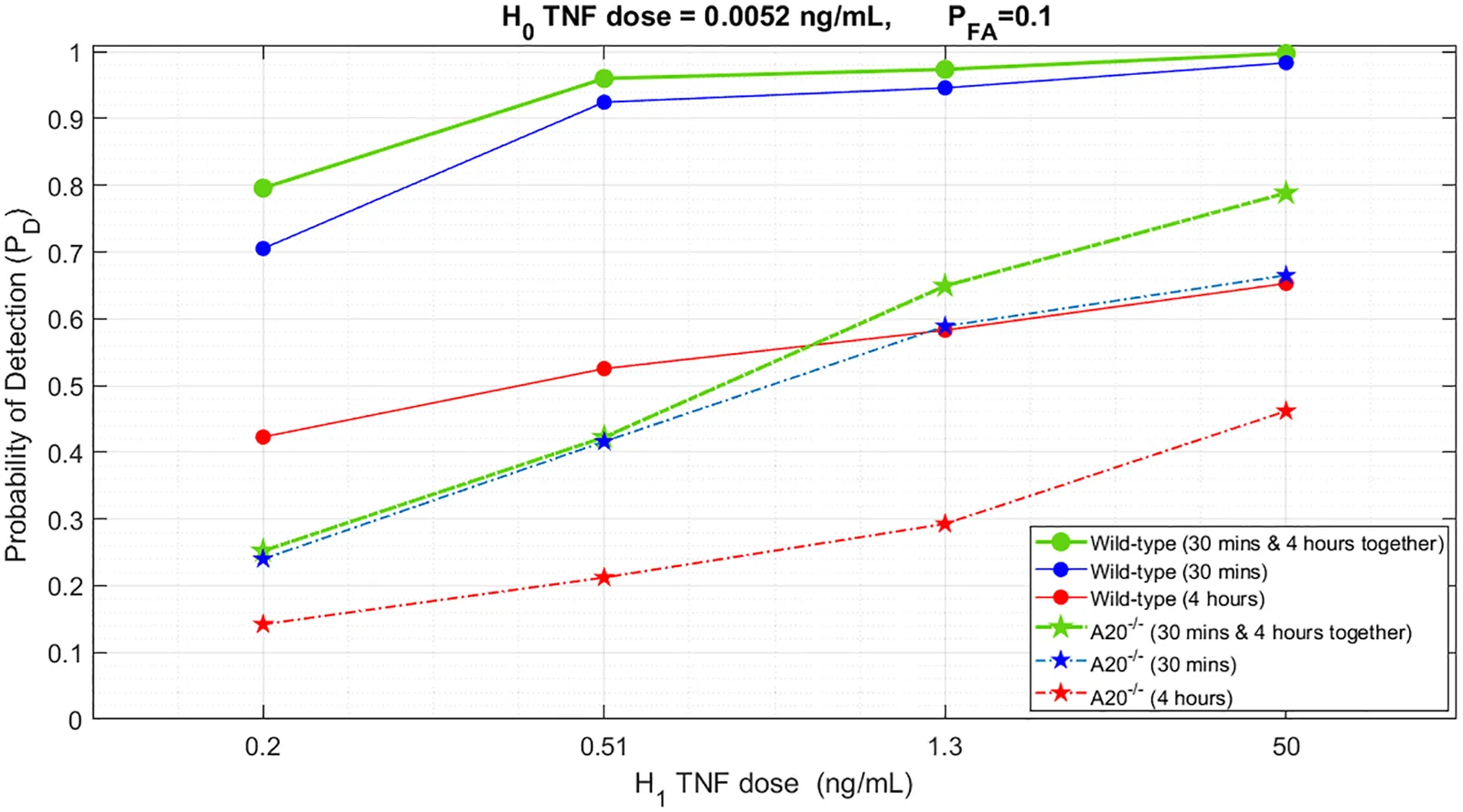
Probability of Detection vs. various TNF levels for both wild-type and A20^−/−^ cells at different time points.

To better sense the excellence of bivariate analysis against univariate analysis in our cellular decision-making framework, Fig 5 is illustrated in which for a fixed *P*_FA_ = 0.1, where low TNF is 0.0052 ng/mL, the probability of detection *P*_D_ for several high TNF levels is graphed, e.g., for wild-type cells, the bivariate *P*_D_(30 minutes & 4 hours) = 0.8, while the individual *P*_D_(30 minutes) = 0.71 and *P*_D_(4 hours) = 0.42 (Table 1). Another noteworthy impression we mentioned above is more obvious here when we compare the wild-type and A20^−/−^ cases for each high TNF levels, e.g., for the high TNF level of 0.2 ng/mL, the *P*_D_ drop is noticeable for individual time point comparison, *P*_D_(wild-type at 30 minutes) = 0.71 while *P*_D_(A20^−/−^ at 30 minutes) = 0.24. When it comes to 4-hour analysis, we can notice the same behavior, *P*_D_(wild-type at 4 hours) = 0.42 while *P*_D_(A20^−/−^ at 4 hours) = 0.14 (Table 1). Besides that, Table 2 shows the same behavior when the high TNF level is 50 ng/mL vs. the same low TNF of 0.0052 ng/mL. The value *P*_FA_ = 0.1 is used throughout as a somewhat conventional engineering reference (analogous to a 5% Type-I error in classical statistics), not a biologically privileged choice; the WT-versus-A20^−/−^ ROC curve ordering is preserved across all values of *P*_FA_ ∈ {0.01, 0.05, 0.10, 0.20, 0.50} (S3 Fig, S1 Table).

These analyses are also done for another two different low TNF levels, 0.0021 ng/mL and 0.013 ng/mL, vs. the aforementioned high TNF levels, 0.2 ng/mL and 50 ng/mL, whose results are all in good agreement.

The corresponding ROCs, along with their eigenvalue decompositions to be used in the Gil-Pelaez lemma for decision-making analysis, are fully provided in SUPPLEMENTARY MATERIALS. These findings provide additional validation of the significance of this parameter within the system model, as we observed biologically meaningful outcomes.

Throughout, the *P*_D_ values in Tables 1 and 2 are reported with bootstrap 95% confidence intervals (CI) obtained from 1000 resamples of the single-cell data.

### Comparison with maximum likelihood detection

The Neyman–Pearson (NP) framework used here differs from the maximum likelihood (ML) approach of our prior work in the statistical parameter or objective to be optimized by the decision-making system or cell. The equal-prior ML rule decides H₁ when the likelihood ratio exceeds 1, yielding a single operating point with a minimized average decision error probability *P*_E_ = 0.5 (*P*_FA_ + *P*_M_), where *P*_M_ = 1 - *P*_D_ is the probability of miss detection. The NP rule instead fixes *P*_FA_ at a chosen level that results in a specific threshold for the likelihood ratio, which in turn reports a maximized *P*_D_, and by varying *P*_FA_, the entire ROC curve can be constructed. The ML operating point can be viewed on a ROC curve (S4 Fig). For TNF of 0.0052 vs. 50 ng/mL at 30 minutes, for example, the ML operating point lies at (*P*_FA_, *P*_D_) = (0.03, 0.97) for wild-type cells but at (0.21, 0.81) for A20^−/−^ cells — i.e., different false alarm rates yet minimized average error rate *P*_E_ of 0.03 and 0.2, respectively. On the other hand, if for example *P*_FA_ of 0.10 is of interest, the NP rule holds *P*_FA_ at 0.10 and results in maximized *P*_D_ of 0.99 and 0.67, respectively, while offering non-minimized average error rate *P*_E_ of 0.055 and 0.215, respectively. Overall, when it comes to the decision performance, neither rule is necessarily superior as each one optimizes a different decision goal.

### Analysis of mixed cell populations

To further demonstrate the biological relevance of the framework, we analyzed a scenario motivated by cancer biology, where malignancies can contain heterogeneous populations of cells with varying A20 expression. We modeled a mixed population in which a fraction β are A20^−/−^ cells and (1 − *β*) are wild-type, with both subpopulations exposed to either low (H₀: 0.0052 ng/mL) or high (H₁: 50 ng/mL) TNF — i.e., under each hypothesis the population is the same (1 − *β*) WT + *β* A20^−/−^ blend, differing only in the TNF level. Because A20^−/−^ cells mount elevated NF-κB responses even under low TNF, mixing the two genotypes erodes a population-level observer’s ability to distinguish low- from high-TNF conditions, most severely at intermediate fractions (quantified below). We emphasize that this is a statistical property of the population readout, not a claim that any individual cell ‘misinterprets’ the signal — single-cell commitment to apoptosis or proliferation is a separate question.

**Fig 6:**
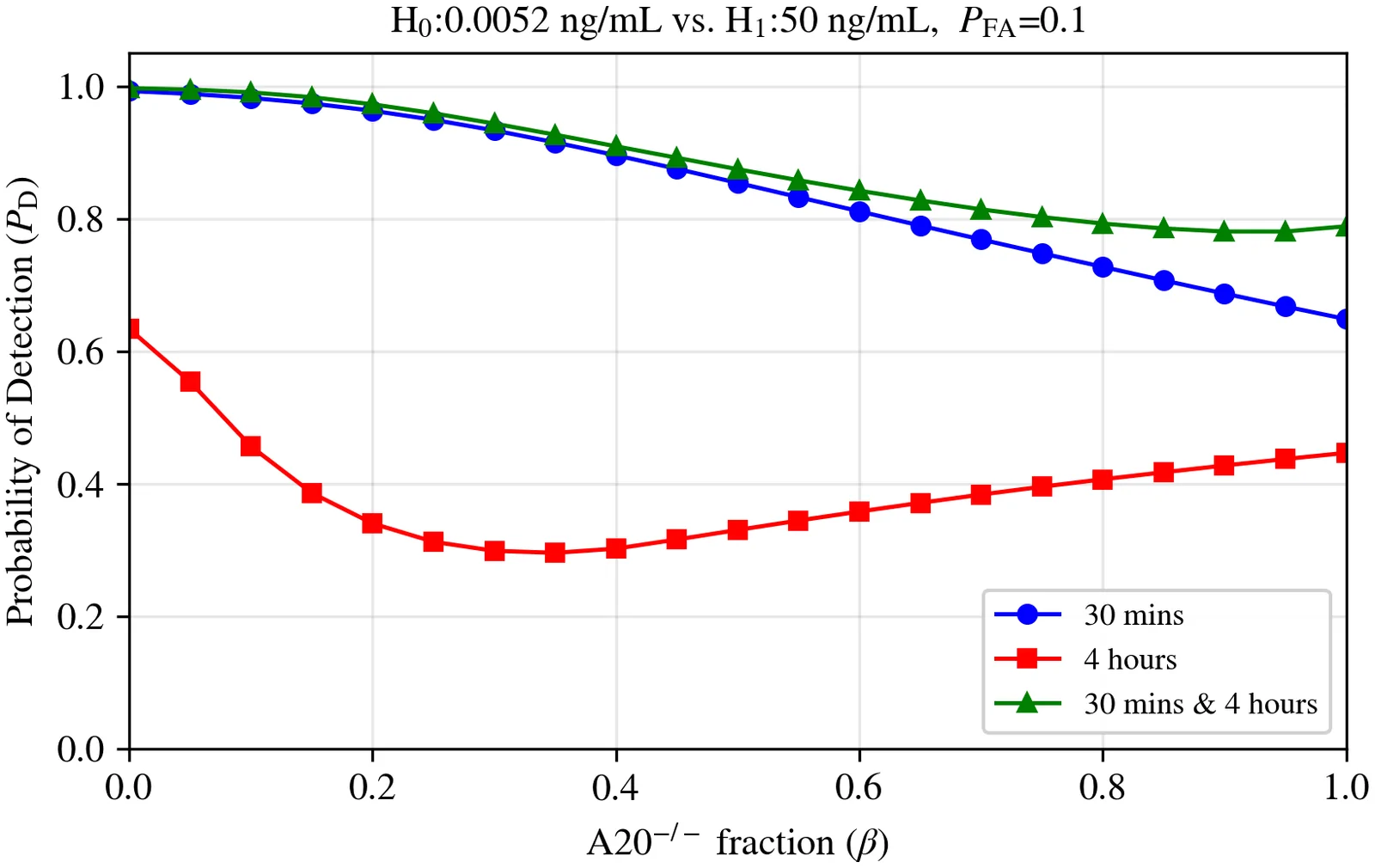
Probability of Detection *P*_D_ at *P*_FA_ = 0.1 versus A20^−/−^ fraction *β* for a mixed population (H_₀_ = 0.0052 vs. H_₁_ = 50 ng/mL), using a single shared (population-observer) Neyman–Pearson threshold. At 4 hours *P*_D_ is non-monotonic, with a minimum near *β* ≈ 0.3.

Fig 6 shows the detection probability *P*_D_ of the optimal population observer — a single shared Neyman–Pearson threshold applied to the mixed-population readout — at *P*_FA_ = 0.1 versus the A20^−/−^ fraction *β*. At the boundaries the results match our pure-population findings: for *β* = 0 (WT), *P*_D_ = 0.99 (30 min), 0.65 (4 h), ≈ 1 (bivariate); for *β* = 1 (A20^−/−^), *P*_D_ = 0.67, 0.46, 0.79 (Table 2). Between these limits the behavior is not monotonic. At 30 minutes *P*_D_ declines smoothly with *β*. At 4 hours *P*_D_ falls steeply to a minimum of ≈0.30 near *β* ≈ 0.3 and then partially recovers toward the pure-A20^−/−^ value as *β* → 1, because at intermediate mixtures the elevated low-TNF (H₀) baseline of A20^−/−^ cells overlaps the high-TNF (H₁) responses of wild-type cells, which a single shared threshold cannot separate; once the population is homogeneous again (*β* → 1) the problem simplifies and *P*_D_ rises. The bivariate curve shows only a shallow version of this dip and remains the highest of the three across the whole range of *β*.

**Fig 7:**
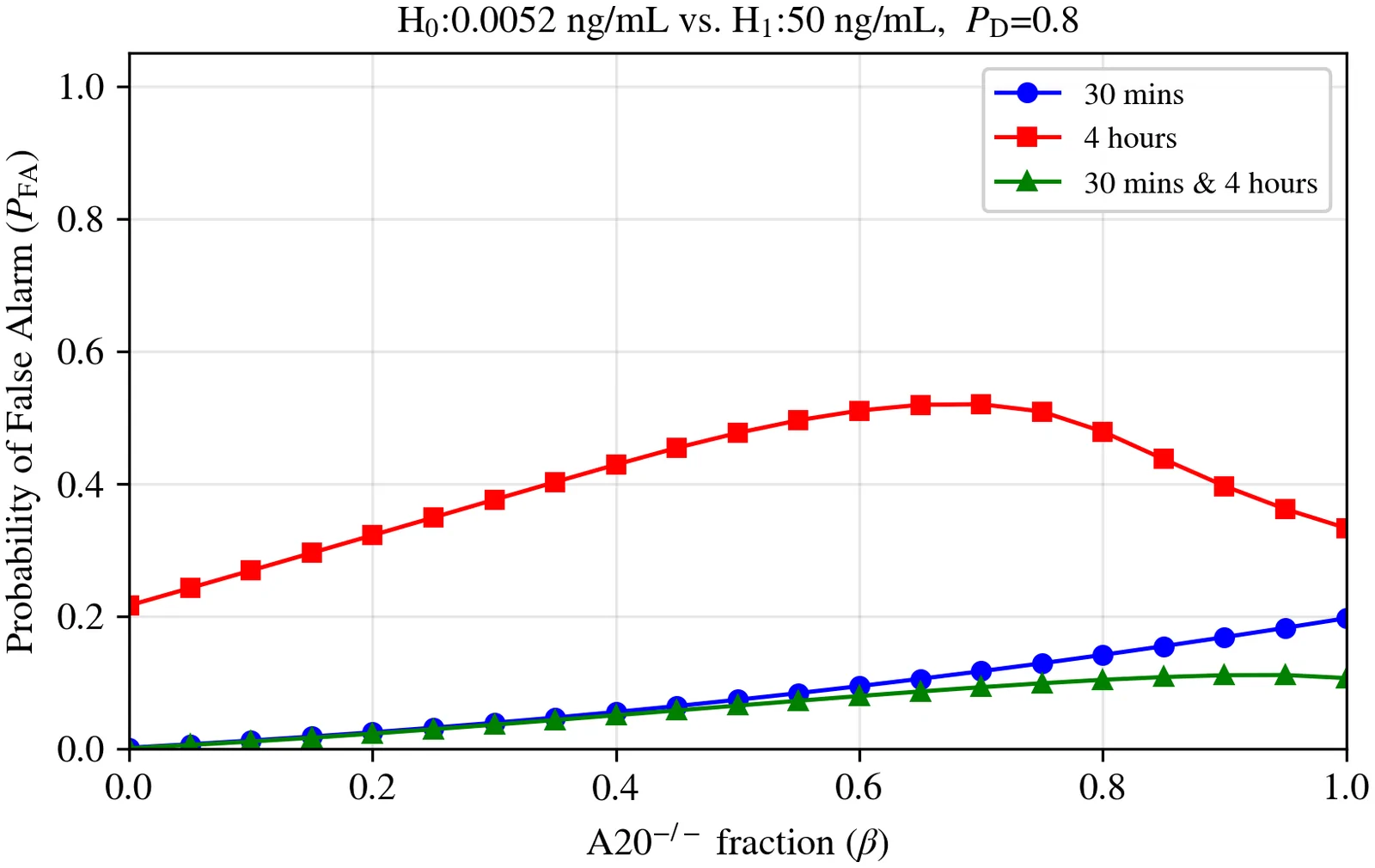
Probability of False Alarm *P*_FA_ at *P*_D_ = 0.8 versus A20^−/−^ fraction *β* for the same mixed population and single shared threshold; the 4-hour curve peaks at intermediate *β*, mirroring Fig 6.

Fig 7 presents the complementary view — the *P*_FA_ required to reach *P*_D_ = 0.8 — as *β* increases. Mirroring Fig 6, the 4-hour curve is non-monotonic: the false alarm cost peaks near *β* ≈ 0.65, where the mixed-population detection is hardest, then declines as *β* → 1. The combined 30-minute-and-4-hour analysis (green) keeps the false alarm rate the lowest of the three across the entire range — roughly half that of the best single time point at high *β* — confirming the robustness of multi-time-point detection in heterogeneous populations.

Figs 6 and 7 adopt the perspective of an external observer applying a single, shared threshold to the mixed population — the biologically pertinent case, since a mutated A20^−/−^ cell cannot recalibrate its own decision threshold but inherits the setpoint of the normal downstream machinery; this inability to reset the threshold is itself a route by which such cells fail to commit to apoptosis and over-proliferate. A genotype-informed observer that gives each subpopulation its own optimal threshold dominates the single shared-threshold observer at every *β* (S5 Fig): the two nearly coincide at 30 minutes, where the mixture is close to Gaussian, and diverge sharply at 4 hours, where the genotype-informed test avoids the shared-threshold dip. This requires single-cell genotype information; when that information is available the problem decouples into two ordinary single-population detections, which can only match or exceed the shared-threshold result.

To make the underlying distributions explicit, S6 Fig shows the full Gaussian-mixture response density at representative mixing fractions (*β* = 0.3, 0.5, 0.7), overlaid on the single-Gaussian approximation for both hypotheses at both time points; the mixture is near-Gaussian at 30 minutes but becomes visibly bimodal at 4 hours for intermediate *β*. Figs 6 and 7 use the exact mixture-LLR (GMM) detector derived in METHODS; the single-Gaussian moment-matched approximation tracks it within |Δ*P*_D_| ≤ 0.017 at 30 minutes and ≤ 0.065 at 4 hours across all *β* (S7 Fig).

An assumption of this analysis is that we treat the wild-type and A20^−/−^ responses as an independent linear mixture, without paracrine signal exchange between the two subpopulations. This is biologically appropriate for the prevailing clinical context of A20 loss — B-cell lymphoma [14] — a hematologic malignancy in which cells circulate and are essentially well-mixed in a homogeneous medium, so the dense paracrine coupling characteristic of solid-tumor stroma is largely absent. In settings with substantial paracrine exchange between wild-type and A20^−/−^ cells, such as solid tumors or dense tissue, a coupled model would instead be required — for example, a paracrine-feedback term modifying the effective TNF input experienced by the A20^−/−^ cells [15] — which we leave to future work.

## Discussion

The introduced approach in this work proposes a set of statistical signal processing and decision-theoretic methods and metrics to model and measure the cellular decision-making errors in a TNF–NF-κB signaling system, under normal conditions and under abnormalities caused by noise or signaling uncertainties. This study showed biologically relevant and meaningful results that agreed with the functionality of the above-mentioned signaling pathway. The important transcription factor NF-κB regulates many genes, controls cell fate, and is involved in apoptosis and the transition of an antiviral state. Therefore, it is crucial for the cell to make an accurate decision in the information transmission of TNF–NF-κB signaling pathway. Here, we focused on such an important cell signaling system for which we presented univariate and bivariate methods to analyze the cell decision-making process and outcomes using Neyman-Pearson theorem, a statistical signal processing method which does not require knowledge of prior probabilities, whereas previous works by Habibi et al., Ozen et al., and Emadi et al. [7–9] require the knowledge of prior probabilities. The signaling pathway could transmit the information of changes in TNF concentration level from 0.0021 ng/mL to 50 ng/mL. The mean estimates in Fig 3A are derived from (and represent) the histograms in Fig 2A. The difference between the means of H_0_ and H_1_ plays a crucial role in the probability of detection *P*_D_ of the proposed model. When the means are further apart, the histograms appear more separated, indicating a higher probability of detection. Conversely, cases with lower *P*_D_ show closer means between H_0_ and H_1_.

A key finding is that when the TNF signal is strong in wild-type cells, the signal detection probability is higher compared to the weak TNF signal detection. The univariate results in Fig 4 demonstrate that in wild-type cells, the system outperforms A20^−/−^ cells in both time points, 30 minutes and 4 hours. Analyzing receiver operating characteristic (ROC), as a measure of system performance, indicates that both time points, 30 minutes and 4 hours, together, which can be considered as a joint or bivariate case, enhances the performance of the decision-making framework. The bivariate case consistently remains either very close to the one with higher *P*_D_ or above both univariate scenarios, the 30 minutes and 4 hours. Furthermore, when comparing the ROC curves from left to right panels (cell transition from wild-type to A20^−/−^ in Figs 4A–4D), a reduction in performance is observed in all cases: 30 minutes, 4 hours, and the bivariate case. This advantage persists even in heterogeneous cell populations containing mixed wild-type and A20^−/−^ cells, demonstrating the robustness of the multivariate approach in biologically realistic scenarios (Figs 6 and 7).

A natural question is whether the optimal decision boundary we derive corresponds to anything a real cell can implement. The boundary is a decision-theoretic optimum — an upper bound on the detectability that any downstream readout can in principle achieve. Whether cells actually approach this bound is an empirical question, and a substantial body of experimental work shows that signaling networks do implement stable decision thresholds, each defined by a dedicated molecular circuit and stable in unperturbed cells, shifting only under specific perturbations such as mutation or pharmacological treatment. In NGF signaling, a curved boundary in the two-dimensional pERK–pAKT plane separates proliferating from differentiating cells and remains invariant across stimuli and upstream perturbations [16]. In the control of cell death, the survival/death decision is set by a threshold in the dedicated apoptotic signaling circuit [17]. In cell migration, distinct migration and polarity patterns are governed by a threshold in the dedicated Rho-GTPase cytoskeletal machinery [18]. And for TNF specifically, the decision of blood-vessel cells to undergo angiogenesis is controlled by a TNF-dose threshold defined by the dedicated angiogenic machinery [15]. In each case the threshold is a stable, reproducible feature of the unperturbed network that shifts predictably only under mutation or drug — exactly the behavior our framework anticipates when such perturbations alter the underlying means and variances. Variability in the downstream network therefore shifts the attainable operating point along the ROC, lowering *P*_D_ at a fixed *P*_FA_, but it does not move the upper bound; the distance between the two is a direct measure of how efficiently a cell’s downstream machinery employs the information available to it.

Because these decision thresholds are set by dedicated downstream networks — the apoptotic and proliferative machinery, largely separate from the upstream signaling cascade — they are typically stable whether a cell is healthy or transformed, and a mutation’s consequence depends on which layer it perturbs. A mutation that perturbs signaling alone, as A20 loss largely does by removing a brake on NF-κB, leaves the threshold fixed but shifts the distribution of inputs reaching it; the population is then read against a single shared threshold, and the non-monotonic loss of discriminability we find for mixed wild-type/A20^−/−^ populations (Figs 6 and 7) is a candidate mechanism by which such signaling lesions drive incorrect survival or proliferation decisions. A mutation or drug that instead alters the threshold-defining network itself shifts the threshold, effectively giving the affected subpopulation its own decision boundary; in that regime discriminability improves and the problem reduces to the genotype-informed (two-threshold) case of S5 Fig. Because A20 is a multifunctional node, both modes may operate, so we present the shared-threshold case in the main text and the genotype-informed case in the supplement to cover either possibility.

The developed framework also makes quantitative, testable predictions. Because detectability depends mainly on the mean separation |μ₁ − μ₀| relative to the noise, any perturbation that narrows this separation or inflates the variance should lower *P*_D_ at a fixed *P*_FA_ by an amount computable from the perturbed model parameters alone. Two pharmacological experiments follow directly. First, partial inhibition of IKK in wild-type cells should compress 30-minute discriminability: IKK activity governs the temporal profile of TNF-induced NF-κB activation [19], and because IKK is the kinase driving the response, lowering it pulls the high-TNF (H₁) response toward the unstimulated baseline. In our data the wild-type H₀–H₁ mean gap at 30 min (0.0052 vs 0.2 ng/mL) is 0.32 versus only 0.14 in A20^−/−^ — the latter already compressed by an elevated H₀ baseline (8.32 vs 8.09) from the missing A20 brake on basal NF-κB — so titrated IKK inhibition should drive the wild-type 30-min ROC toward the A20^−/−^ curve. Second, restoring A20 activity in A20^−/−^ or A20-low cells should rebuild late-phase negative feedback, since A20 terminates NF-κB signaling by editing the ubiquitin chains on RIP1 and targeting it for proteasomal degradation [20]; in our data the A20^−/−^ 4-hour means are elevated under both hypotheses (H₀ 8.54 vs 7.98; H₁ 8.83 vs 8.32), with the larger shift at H₀, so restoring feedback should widen the H₀–H₁ gap and partially rescue the 4-hour detection deficit (*P*_D_ from 0.46 toward the wild-type 0.65 at *P*_FA_ = 0.10). Each prediction is a quantitative ROC shift, not a qualitative expectation, and can be checked by re-fitting the Gaussian parameters after perturbation in new experiments.

The NP framework and the Bayesian/ML approach used previously [7–9] are complementary rather than competing. Both rest on the same likelihood ratio test statistic; they differ in how the decision threshold is chosen, to achieve a different decision objective. The Bayesian/ML route requires committing to the prior probabilities *P*(H₀) and *P*(H₁) which are assumed equal in the earlier studies, and yields a single operating point, whereas the NP route varies the false alarm rate to construct the entire ROC curve without invoking any prior (See also the Comparison with Maximum Likelihood Detection subsection in RESULTS). When the priors are genuinely unknown, as in most single-cell settings, NP provides an assumption-free score. When a prior is meaningfully known — for example a defined experimental ratio of stimulated to unstimulated cells, or an evolutionarily encoded expectation — the appropriate choice is dictated by the decision criterion to be optimized.

There are some limitations to consider. The current analysis assumes that the log-transformed NF-κB responses follow Gaussian distributions, which, while a reasonable approximation supported by the data, may not hold for all signaling systems or under all conditions. Although normality tests reject strict Gaussianity at the large single-cell sample sizes available here [21], the resulting deviations do not practically affect the detection-related results and conclusions: a non-parametric KDE-based detector reproduces the lognormal-based *P*_D_ values to within |Δ*P*_D_| ≤ 0.092 (median |Δ*P*_D_| of 0.03), across all the 48 decision pairs (S2B Table). This is expected because detection performance at a fixed false alarm rate is governed by the ordering the decision statistic imposes on the observations — the ROC is invariant to strictly monotone transformations of that statistic [22,23] — so a good lognormal fit yields nearly the optimal ordering, and the KDE comparison confirms that *P*_D_ is essentially unchanged. Additionally, the framework was validated using a single pathway (TNF–NF-κB) with two time points; extending it to higher-dimensional readouts with more time points or multiple downstream reporters would further demonstrate its generality. Future work could apply this framework to other signaling pathways and disease-relevant contexts, such as characterizing how specific oncogenic mutations degrade signal detection, or integrating it with information-theoretic measures to provide a more complete picture of cellular decision-making fidelity.

The logic of the framework is independent of the number of jointly analyzed readouts *N*; what change with *N* are the computational and statistical costs. Computationally, the Gil-Pelaez lemma remains tractable for *N* ≥ 3 via the characteristic function [24], requiring an *N* × *N* eigen decomposition of the class covariance matrices **Σ** and **Θ**; this is numerically stable for *N* up to ≈ 10 with the covariance matrix condition numbers seen in our data (maximum 1.63; S3 Table). Anyway, the Monte-Carlo quantile estimation introduced for the mixture analysis avoids the eigen decomposition altogether. With regard to the statistical aspect, we note that reliable estimation of an *N* × *N* covariance matrix requires roughly *n* ≥ 10*N* samples [25]. With our per-condition sample sizes (*n* ≈ 137–671), *N* ≈ 5 is a safe number; regularized estimators such as Ledoit–Wolf shrinkage or the graphical lasso extend this to *N* ≈ 20–30 at modest sample sizes. These are the costs of scaling; the benefit side is equally quantifiable. Each additional readout carries an experimental cost — reporter lines, immunostaining channels, or sequencing depth — but because adding a readout can increase the information available to the detector, the framework can calculate the amount of *P*_D_ increase with *N*. This makes NP method a tool for experimental design: starting with a small *N*, one can compute the marginal increase in *P*_D_ at fixed *P*_FA_ from the (*N + 1*)-th readout and stop adding readouts once that gain falls below a chosen threshold.

These findings are also consistent with biological experiments, where A20 deficiency can potentially lead to chronic inflammation or even contribute to the development of cancer. Overall, the introduced decision-theoretic model is particularly suited to settings where the stimulus prior probabilities are unknown — often, though not always, the biological case; and whenever priors are known, it is complementary to a Bayesian/ML analysis, with which it shares the underlying likelihood ratio test.

## Methods

### Experimental data

The data set is composed of wild-type and A20^−/−^ (A20-deficient) 3T3-immortalized mouse embryonic fibroblasts, which can also be labeled as normal and abnormal cells, respectively. Nuclear NF-κB concentrations were measured using immunocytochemistry of thousands of mouse fibroblasts exposed to various TNF levels [26]. Further details of the method used are discussed in [27].

### Neyman-Pearson decision framework

To enhance comprehension of the proposed model in molecular biology and cellular decision-making processes, we applied statistical signal processing and decision theory approaches to analyze the TNF–NF-κB signaling system. A cell is able to decide whether the TNF concentration is low or high when exposed to varying levels of TNF as an input signal [5]. However, due to potential signaling abnormalities or noise in signal transduction, the cell may respond differently to the same input, leading to incorrect decision-making. To address this challenge, we pose a binary hypothesis testing problem in which the cell should determine the true hypothesis based on the TNF input level [7].


$$\begin{array}{c} \left\{ \begin{array}{c} @l@\begin{array}{c} @l{\text{H}}_{\text{0}}\text{:\textbackslash{}hspace\{0.17em}\;Low\;\;TNF\;\;Level,\} \end{array}\mathrm{\backslash vspace}1mm\\ {\text{H}}_{\text{1}}\text{:\textbackslash{}hspace\{0.17em}\;High\;\;TNF\;\;Level.\} \end{array}\right. \end{array}$$ (1)

In the previous works by Habibi et al. [7], Ozen et al. [8], and Emadi et al. [9] the focus was on minimizing the average of the probabilities of the erroneous decisions that a cell can make, *P*_FA_ and *P*_M_ = 1 – *P*_D_ as the statistical metric of interest, where the first one corresponds to the probability of when a cell incorrectly determines that the input level of TNF is high when it is actually low, and the second one refers to the probability of when a cell overlooks a high level of TNF at the input. The optimal decision-making based on the ML approach needed a knowledge of the prior probabilities of the hypotheses H_0_ and H_1_, *P*(H_0_) and *P*(H_1_), as a prerequisite to determine the optimal decision threshold and compute the error probabilities. But typically, in cell signaling studies, such information is unavailable; consequently, they were fairly assumed equal, *P*(H_0_) = *P*(H_1_) = 0.5 in previous works [7–9]. But in this study, we introduce a new approach, based on the Neyman-Pearson theorem, as an alternative approach for cellular decision-making studies which does not ever rely on prior knowledge of the probabilities of the hypotheses H_0_ and H_1_, *P*(H_0_) and *P*(H_1_), and aims to maximize the probability of detection *P*_D_, meaning the probability of identifying the presence of a signal when it does in fact exist [28].

Let *P*_FA_, probability of false alarm, and *P*_D_, probability of detection, be the metric decision probabilities of interest for a cell. *P*_FA_ is indeed the probability of deciding the high level of TNF at the input of the system while it is low (i.e., declaring H_1_ while H_0_ is true), and *P*_D_ is the probability of deciding the high level of TNF when in fact, it is high (i.e., declaring H_1_ while H_1_ is true), as shown below:


$$\begin{array}{c} \begin{array}{c} P_{\text{FA}}=P\left(\text{deciding\textbackslash{}hspace\{0.17em}\;H{\}}_{\text{1}}\text{\textbackslash{}hspace\{0.17em};\;H{\}}_{\text{0}}\right),\mathrm{\backslash vspace}1mm\\ P_{\text{D}}=P\left(\text{deciding\textbackslash{}hspace\{0.17em}\;H{\}}_{\text{1}}\text{\textbackslash{}hspace\{0.17em};\;H{\}}_{\text{1}}\right). \end{array} \end{array}$$ (2)

The optimal decision-making framework strives to maximize *P*_D_ for any given *P*_FA_. To better understand how it works, let vector **y**, the *N*-element observation data (as discussed later, **y** is composed of NF-κB responses), be used to form the likelihood ratio *L*(**y**) in Equation(3). It declares hypothesis H_1_ when the likelihood ratio *L*(**y**) is greater than the threshold γ, and declares H_0_ otherwise, according to the Neyman-Pearson theorem [28–30].


$$\begin{array}{c} L\left(𝐲\right)=\frac{p(𝐲;{\text{H}}_{\text{1}})}{p(𝐲;{\text{H}}_{\text{0}})}>\gamma \text{\textbackslash{}hspace\{0.17em}\},\text{\textbackslash{}hspace\{0.17em}\;\;\;\;Decides\;\;H{\}}_{\text{1}}\text{.} \end{array}$$ (3)

To further analyze the performance of the proposed framework, we need to compute the optimal decision threshold. First step is to define the metric parameters of interest, probability of false alarm *P*_FA_, and probability of detection *P*_D_ which are computed as follows:


$$\begin{array}{c} \underset{\{𝐲\in \text{\textasciitilde{}False\textasciitilde{}Alarm\textasciitilde{}Region}\}}{P_{\text{FA}}=\int ...\int p(𝐲\text{\textasciitilde{}};{\text{\textasciitilde{}H}}_{\text{0}})\text{\textasciitilde{}}d𝐲\text{\textasciitilde{}}=\text{\textasciitilde{}}\alpha }, \end{array}$$ (4)

$$\begin{array}{c} \underset{\text{\textasciitilde{}}\{𝐲\in \text{\textasciitilde{}Detection\textasciitilde{}Region}\}}{P_{\text{D}}=\int ...\int p(𝐲\text{\textasciitilde{}};{\text{\textasciitilde{}H}}_{\text{1}})\text{\textasciitilde{}}d𝐲}. \end{array}$$ (5)

where *p*(**y**; H_0_) and *p*(**y**; H_1_) are the probability density functions (PDFs) of **y** under hypotheses H_0_ and H_1_, respectively. There is a specific γ value to be calculated for any given *P*_FA_ equal to α in Equation (4) by which the maximized *P*_D_ can be obtained as shown in Equation (5). Upon obtaining these decision probabilities, we can also take advantage of a well-known graphical representation, the Receiver Operating Characteristic (ROC), to visualize the performance of the proposed decision-making framework (Fig 4).

The utilized experimental data were first modeled using lognormal PDFs, for mathematical convenience and also to gain insights. In other words, the natural logarithm of data, ln(data), was first considered to be Gaussian. The lognormal model was initially examined by formal goodness-of-fit analysis (Shapiro–Wilk, Anderson–Darling and Jarque–Bera tests; S2A Table) and quantile–quantile (Q–Q) plots (S8 Fig). Then the usefulness of the lognormal model in the detection context was verified by showing that a non-parametric kernel density estimator (KDE) applied to the raw single-cell data reproduced the lognormal *P*_D_ values to within |Δ*P*_D_| ≤ 0.092 (median |Δ*P*_D_| of 0.03), across all the 48 decision pairs (S2B Table). The multivariate Gaussian PDF of the measured response quantity of data, for **y** under the hypothesis H_*i*_ is shown below [28]:


$$\begin{array}{c} p(𝐲\text{\textbackslash{}hspace\{0.17em}\};\text{\textbackslash{}hspace\{0.17em}H{\}}_{i})=\frac{\text{1}}{(2\pi {)}^{N/2}{\left|\Sigma_{i}\right|}^{1/2}}exp\left[-\frac{\text{1}}{\text{2}}(𝐲-\mu_{i}{)}^{T}\Sigma_{i}^{-1}(𝐲-\mu_{i})\right],\text{\textbackslash{}hspace\{0.17em}\;\;\;\}i=0,1. \end{array}$$ (6)

where **y** represents the N×1 column vector of the ln(data), $\mu_{i}$ is the N×1 mean vector of **y** under hypothesis H_*i*_ and $\Sigma_{i}$ is the N×N covariance matrix of y under hypothesis H_*i*_. $\left|\Sigma_{i}\right|$ and ${\Sigma_{i}}^{-1}$ demonstrate the determinant and inverse of the matrix $\Sigma_{i}$, respectively. And ^*T*^ denotes the transpose operator.

As mentioned earlier, to assess the performance of the proposed framework, the optimal decision threshold and the probabilities of interest in Equations (4) and (5) need to be computed in the following subsections, in a simpler case of univariate, and the general multivariate case.

A **Univariate Decision Analysis**

We use nuclear NF-κB responses of thousands of cells exposed to various TNF concentrations measured at 30 minutes and 4 hours after exposure [5]. For univariate analysis, we consider *y* = ln (nuclear NF-κB) for each time point, individually (for simplicity, we may drop the term “nuclear” in some instances). As mentioned earlier, a Gaussian PDF can well represent the data, so for the case of univariate analysis, when *N* = 1 in Equation (6):


$$\begin{array}{c} p(y;{\text{H}}_{i})=\frac{\text{1}}{(2\pi \sigma_{i}^{\text{2}}{)}^{1/2}}exp\left[\frac{-(y-\mu_{i}{)}^{\text{2}}}{2\sigma_{i}^{\text{2}}}\right],\text{\textbackslash{}hspace\{0.17em}\;\;\;\}i=0,1\text{\textbackslash{}hspace\{0.17em}\}. \end{array}$$ (7)

Where $\mu_{i}$ is the mean and $\sigma_{i}^{\text{2}}$ is the variance of data under *i*-th hypothesis H_*i*_. Under H_0_, TNF concentration levels 0.0021, 0.0052, and 0.013 ng/mL, and under H_1_, TNF concentration levels of 0.2, 0.51, 1.3, and 50 ng/mL were used.

To analyze the univariate decision, first, we need to define the random variable *Y* as below:


$$\begin{array}{c} Y\sim \;\left\{ \begin{array}{c} @c@\begin{array}{c} @lN\text{(}\mu_{\text{0}},\sigma_{\text{0}}^{\text{2}})\text{\textbackslash{}hspace\{0.17em}\;\;\;\;\;\;\;\;\;\;\;\;\;H{\}}_{\text{0}}, \end{array}\\ N\left(\mu_{\text{1}},\sigma_{\text{1}}^{\text{2}}\right)\text{\textbackslash{}hspace\{0.17em}\;\;\;\;\;\;\;\;\;\;\;\;\;H{\}}_{\text{1}}. \end{array}\right.\text{\textbackslash{}hspace\{0.17em}\;\;\;\;\;\} \end{array}$$ (8)

Where N stands for the normal (Gaussian) PDF. Equation (8)indicates that the random variable *Y* under hypothesis H_0_ has a normal distribution with mean $\mu_{\text{0}}$ and variance $\sigma_{\text{0}}^{\text{2}}$. It also has a normal distribution with mean $\mu_{\text{1}}$ and variance $\sigma_{\text{1}}^{\text{2}}$, under hypothesis H_1_. Then, the transformation $X=(Y-\mu_{\text{0}})/\sigma_{\text{0}}$ is applied to make the normal distribution $N\text{(}\mu_{\text{0}},\sigma_{\text{0}}^{\text{2}})\text{\textbackslash{}hspace\{0.17em}\;\}$ under H_0_, a normal distribution of zero mean and unit variance. This transformation helps us in further simplification of equations and makes analytics more straightforward when it comes to the case of multivariate decision analysis. So, the hypothesis testing problem in Equation (8)for random variable *Y* turns into the new hypothesis testing problem of Equation (9)for *X*, which is actually the new transformed univariate random variable *Y*.


$$\begin{array}{c} X\sim \;\left\{ \begin{array}{c} @c@\begin{array}{c} @lN\text{(0},1)\text{\textbackslash{}hspace\{0.17em}\;\;\;\;\;\;\;\;\;\;\;\;\;H{\}}_{\text{0}}, \end{array}\\ N\left(m,\lambda \right)\text{\textbackslash{}hspace\{0.17em}\;\;\;\;\;\;\;\;\;\;H{\}}_{\text{1}}. \end{array}\right. \end{array}$$ (9)

Where $m=\frac{\mu_{\text{1}}-\mu_{\text{0}}}{\sigma_{\text{0}}}\text{\textbackslash{}hspace\{0.17em}\;and\;\;\;\}\lambda =\frac{\sigma_{\text{1}}^{\text{2}}}{\sigma_{\text{0}}^{\text{2}}}$.

To find the test statistic *Z* (a statistic, as a function of the data), we need to form the likelihood ratio first. As mentioned above, the importance of the transformation above will become more apparent in the next subsection **B**, when we look at *N* > 1, where finding the PDF of the test statistic is rather complicated.

The optimal decision rule declares H_1_, if:


$$\begin{array}{c} L\left(x\right)=\frac{p(x;{\text{H}}_{\text{1}})}{p(x;{\text{H}}_{\text{0}})}=\frac{\frac{\text{1}}{\sqrt{2\pi \lambda }}exp\left(-\frac{(x-m{)}^{\text{2}}}{2\lambda }\right)\text{\textbackslash{}hspace\{0.17em}}{\cdot }\text{\textbackslash{}hspace\{0.17em}\}\}\frac{\text{1}}{\sqrt{2\pi }}exp\left(-\frac{x^{\text{2}}}{\text{2}}\right)\text{\textbackslash{}hspace\{0.17em}\;\;\}>\gamma , \end{array}$$ (10)

to find the optimal decision threshold for *x*, and after further algebraic simplification of the Equation (10), we obtain the following quadratic equation:


$$\begin{array}{c} x^{\text{2}}-\frac{(x-m{)}^{\text{2}}}{\lambda }>\underset{\gamma \text{'}}{\underbrace{2ln\left(\gamma \sqrt{\lambda }\right)}}, \end{array}$$ (11)

so, using the Equation (11), the test statistic can be defined as below:


$$\begin{array}{c} Z=X^{\text{2}}-\lambda^{-1}(X-m{)}^{\text{2}}. \end{array}$$ (12)

It is worth mentioning that the log-likelihood ratio for Gaussian PDFs is quadratic in the observation, the decision region is generally specified by its two roots, and consequently the Neyman-Pearson decision region can comprise two thresholds rather than a single threshold. We compute both roots of the quadratic equation and determine the decision region that attains the target *P*_FA_. In practice, typically one threshold falls in a region of negligible probability mass, reducing the decision rule to a single effective threshold: most of the 48 (cell-type × time × H₀ × H₁) conditions follow this pattern (S4 Table), so the single-threshold approximation is numerically accurate and sufficient for our data. Anyway, for every condition, the reported detection probability is computed using the exact decision region obtained by Monte Carlo, so no single-threshold approximation is made. Also, for the bivariate case (*N* = 2), the *L*(**x**) = *γ* contour forms a continuous boundary in the two-dimensional log-response plane (see our previous study in [9]).

To obtain the optimal decision threshold *Z*_th_ from *P*_FA_ in Equation (4), we use the Cumulative Distribution Function (CDF) of the test statistic *Z*, *F*_*Z*_ (*z*):


$$\begin{array}{c} F_{Z}\left(z\right)=P(Z\leq z)=\int_{-\infty }^{z}p_{Z}\left(u\right)\text{\textbackslash{}hspace\{0.17em}\;du\}, \end{array}$$ (13)

So, the decision probabilities of interest in the Equations (4) and (5) can be calculated using Equation (13), as follows:


$$\begin{array}{c} P_{\text{FA}}=\int_{z_{\text{th}}}^{\infty }p_{Z}(z;{\text{H}}_{\text{0}})\text{\textbackslash{}hspace\{0.17em}dz\}=1-F_{Z}(z_{\text{th}};{\text{H}}_{\text{0}}), \end{array}$$ (14)

$$\begin{array}{c} P_{\text{D}}=\int_{-\infty }^{z_{\text{th}}}p_{Z}(z;{\text{H}}_{\text{1}})\text{\textbackslash{}hspace\{0.17em}dz\}=1-F_{Z}(z_{\text{th}};{\text{H}}_{\text{1}}). \end{array}$$ (15)

where the *F*_Z_ (*z*; H_*i*_) is the CDF of the test statistic *Z* given hypothesis H_*i*_. As discussed earlier, for any given *P*_FA_ = α, the *z*_th_ can be obtained from the Equation (14), but first the according *F*_Z_ (*z*) needs to be calculated.

To calculate *F*_Z_ (*z*) through the Gil-Pelaez lemma [24,31], the characteristic function of the test statistic *Z* is required:


$$\begin{array}{c} F_{Z}\left(z\right)=\frac{\text{1}}{\text{2}}-\frac{\text{1}}{\pi }\int_{\text{0}}^{\infty }Im\left[e^{-j\omega z}\omega^{-1}\varphi_{Z}\left(\omega \right)\right]\text{\textbackslash{}hspace\{0.17em}\;\}d\omega , \end{array}$$ (16)

where the Im[*x*] is the imaginary part of *x* and *j* = $\sqrt{-\text{1}}$.

As mentioned above, the characteristic functions of the test statistic *Z* are needed for computing *P*_FA_ and *P*_D_. Then, $\varphi_{Z}\left(\omega \right)$ under hypothesis H_0_ and H_1_ can be obtained as below:


$$\begin{array}{c} \begin{array}{c} \varphi_{Z}(\omega \text{\textbackslash{}hspace\{0.17em}\};\text{\textbackslash{}hspace\{0.17em}H{\}}_{\text{0}})=E\left[exp\left(j\omega Z\right)\text{\textbackslash{}hspace\{0.17em}\};\text{\textbackslash{}hspace\{0.17em}H{\}}_{\text{0}}\right]=E\left[exp(j\omega \text{\textbackslash{}hspace\{0.17em}\}(X^{\text{2}}-\lambda^{-1}(X-m{)}^{\text{2}}))\text{\textbackslash{}hspace\{0.17em}\};\text{\textbackslash{}hspace\{0.17em}H{\}}_{\text{0}}\right],\\ =\underset{-\infty }{\int^{+\infty }}exp(j\omega \text{\textbackslash{}hspace\{0.17em}\left(x^{\text{2}}-\lambda^{-1}(x-m{)}^{\text{2}}\right))\}\text{\textbackslash{}hspace\{0.17em}\}.\text{\textbackslash{}hspace\{0.17em}\}\frac{\text{1}}{\sqrt{2\pi }}exp(-\frac{x^{\text{2}}}{\text{2}})\text{\textbackslash{}hspace\{0.17em}\;\}dx,\\ =\text{\textbackslash{}hspace\{0.17em}\}{\left(1-\frac{j2\omega \text{\textbackslash{}hspace\{0.17em}}{(}\lambda -1)\}\lambda \right)}^{-1/2}\text{\textbackslash{}hspace\{0.17em}\}exp\left({\frac{\omega \text{\textbackslash{}hspace\{0.17em}}{m}}^{\text{2}}(1-j2\omega )\}j\lambda +2\omega \text{\textbackslash{}hspace\{0.17em}(\lambda -1)\}\right), \end{array} \end{array}$$ (17)

$$\begin{array}{c} \begin{array}{c} \varphi_{Z}(\omega \text{\textbackslash{}hspace\{0.17em}\};\text{\textbackslash{}hspace\{0.17em}H{\}}_{\text{1}})=E\left[exp\left(j\omega Z\right)\text{\textbackslash{}hspace\{0.17em}\};\text{\textbackslash{}hspace\{0.17em}H{\}}_{\text{1}}\right]=E\left[exp(j\omega \text{\textbackslash{}hspace\{0.17em}\}(X^{\text{2}}-\lambda^{-1}(X-m{)}^{\text{2}}))\text{\textbackslash{}hspace\{0.17em}\};\text{\textbackslash{}hspace\{0.17em}H{\}}_{\text{1}}\right],\\ =\underset{-\infty }{\int^{+\infty }}exp(j\omega \text{\textbackslash{}hspace\{0.17em}\left(x^{\text{2}}-\lambda^{-1}(x-m{)}^{\text{2}}\right))\}\text{\textbackslash{}hspace\{0.17em}\}.\text{\textbackslash{}hspace\{0.17em}\}\frac{\text{1}}{\sqrt{2\pi \lambda }}exp(-\frac{(x-m{)}^{\text{2}}}{2\lambda })\text{\textbackslash{}hspace\{0.17em}\;\}dx,\\ =\text{\textbackslash{}hspace\{0.17em}\}{\left(1-j2\omega \text{\textbackslash{}hspace\{0.17em}\}(\lambda -1)\right)}^{-1/2}\text{\textbackslash{}hspace\{0.17em}\}exp\left(-{\frac{\omega \text{\textbackslash{}hspace\{0.17em}}{m}}^{\text{2}}(1+j2\omega )\}j+2\omega \text{\textbackslash{}hspace\{0.17em}(\lambda -1)\}\right). \end{array} \end{array}$$ (18)

Here, *E*[*x*] denotes the expected value of *x*. This route is especially useful when *N* > 1. In principle, one could obtain *F*_Z_(*z*) by integrating the PDF *p*_*z*_(*z*), but for our test statistic *Z*, the PDF has no simple closed form when *N* > 1 (see subsection B). Instead, we compute *F*_Z_(*z*) numerically by using the characteristic function from Equations (17) and (18) together with the Gil-Pelaez inversion in Equation (16). With *F*_Z_(*z*) in hand, *P*_FA_ and *P*_D_ can be computed using Equations (14) and (15).

B **Multivariate Decision Analysis:**

In the general case, we further expand the study to multivariate decision analysis when *N* > 1, e.g., when *N* = 2, the decision probabilities of interest when NF-κB responses are analyzed for the concurrent case of two different time points of 30 minutes and 4 hours after TNF exposure in the RESULTS section. To compute the *P*_FA_ and *P*_D_ for this case, we need to take all the steps of univariate decision analysis (subsection **A**). First, for the *N*-element vector **Y** below, we use the multivariate Gaussian PDF of Equation (6) to form the likelihood ratio for the hypothesis testing problem in Equation (19). So, for the multivariate random vector **Y**, we have:


$$\begin{array}{c} \begin{array}{c} 𝐘\sim \;\left\{ \begin{array}{c} @c@\begin{array}{c} N(\mu ,\Sigma )\text{\textbackslash{}hspace\{0.17em}\;\;\;\;\;\;\;\;\;\;\;\;H{\}}_{\text{0}} \end{array}\\ N\left(\eta ,\Theta \right)\text{\textbackslash{}hspace\{0.17em}\;\;\;\;\;\;\;\;\;\;\;\;\;H{\}}_{\text{1}} \end{array}\right.\text{\textbackslash{}hspace\{0.17em}\;\;\;\;\;\;\;\;\;\;\;\;\;\;\;\;\;\;\;\;\;\;\;\}𝐘=\left[\begin{array}{c} Y_{\text{1}}\\ \text{\textbackslash{}hspace\{0.17em}\;\}.\\ \text{\textbackslash{}hspace\{0.17em}\;\}.\\ \text{\textbackslash{}hspace\{0.17em}\;\}.\\ Y_{N} \end{array}\right]\text{\textbackslash{}hspace\{0.17em}\},\text{\textbackslash{}hspace\{0.17em}\;\;\;\;\;\;\;\;\;\;\;\;\;\;\}\begin{array}{c} \begin{array}{c} \mu =\left[\begin{array}{c} \mu_{\text{1}}\\ \text{\textbackslash{}hspace\{0.17em}\;\}.\\ \text{\textbackslash{}hspace\{0.17em}\;\}.\\ \text{\textbackslash{}hspace\{0.17em}\;\}.\\ \mu_{N} \end{array}\right], \end{array}\\ \eta =\left[\begin{array}{c} @l@\eta_{\text{1}}\\ \text{\textbackslash{}hspace\{0.17em}\;\}.\\ \text{\textbackslash{}hspace\{0.17em}\;\}.\\ \text{\textbackslash{}hspace\{0.17em}\;\}.\\ \eta_{N} \end{array}\right], \end{array}\text{\textbackslash{}hspace\{0.17em}\;\;\;\;\;\;\;\;\;\;\;\;\;\;\;\;\}\begin{array}{c} \begin{array}{c} \Sigma =E\left[\left(𝐘-\mu \right){\left(𝐘-\mu \right)}^{T}\right], \end{array}\\ \Theta =E\left[\left(𝐘-\eta \right){\left(𝐘-\eta \right)}^{T}\right], \end{array} \end{array} \end{array}$$ (19)

where μ is the N×1 mean vector and Σ is the N×N covariance matrix under hypothesis H_0_. Also, η is the N×1 mean vector and Θ represents the N×N covariance matrix under hypothesis H_1_. Note that these mean vectors and covariance matrices are the same as those in Equation (6), however they are introduced here to make the notation simpler for the multivariate formulation and analysis.

In the bivariate case considered here (*N* = 2), where the two components of **Y** are cell’s 30-minute and 4-hour readouts, both intrinsic noise and extrinsic noise — for example cell-to-cell variation at receptor or pathway-component levels — are absorbed into the estimated mean vectors μ and η, and covariance matrices Σ and Θ, and therefore integrated into the decision test statistic. Each off-diagonal entry of each covariance matrix captures the correlation between a cell’s 30-minute and 4-hour responses — if extrinsic fluctuations induced such cross-time correlation, the bivariate likelihood ratio test would exploit it. In the present dataset, however, these two readouts come from independent fixed-cell populations with no tracking of individual cells across the two time points. For experiments that do track single cells over time, such as live-cell imaging, the developed decision test statistic is capable of capturing and taking into account any possible cross-time correlations.

As discussed earlier, to normalize the PDF under H_0_, making it a zero-mean and unit-variance Gaussian PDF, we do a transformation that obviously affects the PDF under H_1_ as well. First, we start with an eigenvalue decomposition of Σ, the covariance matrix under hypothesis H_0_ as follows [28]:


$$\begin{array}{c} \Sigma =𝐔\text{\textbackslash{}hspace\{0.17em}\}𝐃\text{\textbackslash{}hspace\{0.17em}\}{𝐔}^{T}, \end{array}$$ (20)

where **U** is the orthonormal matrix of eigenvectors, and **D** is the diagonal matrix of eigenvalues of the covariance matrix Σ. The vector **Y** under the hypothesis H_0_ is transformed through three steps; first, the mean is subtracted to obtain **Y-** μ; second, elements of the zero-mean **Y** are de-correlated according to **U**^*T*^(**Y-** μ); and third, the de-correlated elements are made unit-variance by calculating $\tilde{𝐗}={𝐃}^{-1/2}\text{\textbackslash{}hspace\{0.17em}\}{𝐔}^{T}\text{\textbackslash{}hspace\{0.17em}\}(𝐘-\mu ),$ where ${𝐃}^{-1/2}$ is the diagonal matrix of the square root of the inverse of the eigenvalues. Clearly, upon repeating all the steps mentioned above, the mean vector and covariance matrix of **Y** under the hypothesis H_1_ will be transformed accordingly, as shown below:


$$\begin{array}{c} \to \text{\textbackslash{}hspace\{0.17em}\;\}\tilde{𝐗}\text{ \textbackslash{}hspace\{0.17em}\}\left\{ \begin{array}{c} @l@\begin{array}{c} @lN\text{(}0,𝐈)\text{\textbackslash{}hspace\{0.17em}\;\;\;\;\;\;\;\;\;\;\;\;\;\;\;\;\;\;\;\;\;\;\;\;\;\;\;\;\;\;\;\;\;\;\;\;\;\;\;\;\;\;\;\;\;\;H{\}}_{\text{0}}\text{\textbackslash{}hspace\{0.17em}\} \end{array}\\ N\left({𝐃}^{-1/2}\text{\textbackslash{}hspace\{0.17em}\}{𝐔}^{T}\text{\textbackslash{}hspace\{0.17em}\}\left(\eta -\mu \right),𝐂\right)\text{\textbackslash{}hspace\{0.17em}\;\;\;\;\;\;\;\;\;\;\;\;\;H{\}}_{\text{1}}\text{\textbackslash{}hspace\{0.17em}\} \end{array}\right.\;\;\;\;\;,\text{\textbackslash{}hspace\{0.17em}\;\;\;\;\;\;\;\;\;\;\;\;\;\;\;\}\begin{array}{c} 𝐂={𝐃}^{-1/2}\text{\textbackslash{}hspace\{0.17em}\}{𝐔}^{T}\text{\textbackslash{}hspace\{0.17em}\}\Theta \text{\textbackslash{}hspace\{0.17em}\}𝐔\text{\textbackslash{}hspace\{0.17em}\}{𝐃}^{-1/2}, \end{array} \end{array}$$ (21)

where 𝐂 is the covariance matrix of $\tilde{𝐗}$ under the hypothesis H_1_ in Equation (21), which is determined by the two original covariance matrices. The next step would be applying another transformation to reach the new vector **X**, as the desired transformed version of **Y**. The new covariance matrix **C** of the transformed **Y**, $\tilde{𝐗}$ under hypothesis H_1_ can be decomposed into its eigenvector and eigenvalue matrices as $𝐂=𝐕\text{\textbackslash{}hspace\{0.17em}\}\Lambda \text{\textbackslash{}hspace\{0.17em}\}{𝐕}^{T}$ where **V** is the orthonormal matrix of eigenvectors, and Λ is the diagonal matrix of eigenvalues of the covariance matrix **C**. Then, **X** is constructed by de-correlating $\tilde{𝐗}$ under the hypothesis H_1_ in Equation (21) according to $𝐗={𝐕}^{T}\tilde{𝐗}={𝐕}^{T}\text{\textbackslash{}hspace\{0.17em}\}{𝐃}^{-1/2}\text{\textbackslash{}hspace\{0.17em}\}{𝐔}^{T}\text{\textbackslash{}hspace\{0.17em}\}(𝐘-\mu )$. Therefore, **X** as shown below, has zero-mean and unit-variance uncorrelated Gaussian variables under H_0_, whereas under H_1_ it has uncorrelated Gaussian variables whose means and variances are given by **m** and **Λ**, respectively:


$$\begin{array}{c} \to \text{\textbackslash{}hspace\{0.17em}\;\}𝐗\sim \;\left\{ \begin{array}{c} @c@\begin{array}{c} N\text{(}0,𝐈)\text{\textbackslash{}hspace\{0.17em}\;\;\;\;\;\;\;\;\;\;\;\;\;\;\;\;\;\;\;\;\;\;\;H{\}}_{\text{0}}\text{\textbackslash{}hspace\{0.17em}\;\} \end{array}\\ N\left(𝐦,\Lambda \right)\text{\textbackslash{}hspace\{0.17em}\;\;\;\;\;\;\;\;\;\;\;\;\;\;\;\;\;\;\;H{\}}_{\text{1}}\text{\textbackslash{}hspace\{0.17em}\;\;\} \end{array},\text{\textbackslash{}hspace\{0.17em}\;\;\;\;\;\;\;\;\;\;\;\;\}\right.\text{\textbackslash{}hspace\{0.17em}\;\}𝐗=\left[\begin{array}{c} @l@X_{\text{1}}\\ \text{\textbackslash{}hspace\{0.17em}\;\;\}.\\ \text{\textbackslash{}hspace\{0.17em}\;\;\}.\\ \text{\textbackslash{}hspace\{0.17em}\;\;\}.\\ X_{N} \end{array}\right]\text{\textbackslash{}hspace\{0.17em}\},\text{\textbackslash{}hspace\{0.17em}\;\;\;\;\;\;\;\;\;\;\;\;\;\;\}\begin{array}{c} 𝐦=\left[\begin{array}{c} @l@m_{\text{1}}\\ \text{\textbackslash{}hspace\{0.17em}\;\;\}.\\ \text{\textbackslash{}hspace\{0.17em}\;\;\}.\\ \text{\textbackslash{}hspace\{0.17em}\;\;\}.\\ m_{N} \end{array}\right], \end{array}\text{\textbackslash{}hspace\{0.17em}\;\;\;\;\;\;\;\;\;\;\;\;\;\}\begin{array}{c} \Lambda =\left[\begin{array}{ccc} @ccc@\lambda_{\text{1}} & .\text{\textbackslash{}hspace\{0.17em}\;\}.\text{\textbackslash{}hspace\{0.17em}\;\}. & 0\\ \begin{array}{c} .\\ .\\ . \end{array} & \begin{array}{c} .\\ \text{\textbackslash{}hspace\{0.17em}\;\}.\\ \text{\textbackslash{}hspace\{0.17em}\;\;\;\}. \end{array} & \begin{array}{c} .\\ .\\ . \end{array}\\ 0 & .\text{\textbackslash{}hspace\{0.17em}\;\}.\text{\textbackslash{}hspace\{0.17em}\;\}. & \lambda_{N} \end{array}\right], \end{array} \end{array}$$ (22)

where $𝐦={𝐕}^{T}\text{\textbackslash{}hspace\{0.17em}\}{𝐃}^{-1/2}\text{\textbackslash{}hspace\{0.17em}\}{𝐔}^{T}\text{\textbackslash{}hspace\{0.17em}\}(\eta -\mu )$ is the new mean vector and Λ is the new covariance matrix under the hypothesis H_1_ in Equation (22).

As mentioned earlier, such a transformation can help us in further analytical simplifications. The optimal likelihood-based decision framework for the vector **Y** in Equation (19) decides H_1_ if:


$$\begin{array}{c} L\left(𝐲\right)=\frac{p_{𝐘}(𝐲;{\text{H}}_{\text{1}})}{p_{𝐘}(𝐲;{\text{H}}_{\text{0}})}=\frac{\frac{\text{1}}{(2\pi {)}^{N/2}\text{\textbackslash{}hspace\{0.17em}}{\left|\Theta \right|}^{1/2}}{exp}\left(-\frac{\text{1}}{\text{2}}(𝐲-\eta {)}^{T}\Theta^{-1}(𝐲-\eta )\right)\}\frac{\text{1}}{{\left(2\pi \right)}^{N/2}\text{\textbackslash{}hspace\{0.17em}}{\left|\Sigma \right|}^{1/2}exp\left(-\frac{\text{1}}{\text{2}}(𝐲-\mu {)}^{T}\Sigma^{-1}(𝐲-\mu )\right)\}>\gamma \text{\textbackslash{}hspace\{0.17em}\},\text{\textbackslash{}hspace\{0.17em}\;\;\;\;\;\;\;\;Decide\;\;H{\}}_{\text{1}}\text{\textbackslash{}hspace\{0.17em}\}. \end{array}$$ (23)

But the optimal likelihood-based decision rule for **X**, the transformed variable **Y**, declares H_1_, if:


$$\begin{array}{c} L\left(𝐱\right)=\frac{p_{𝐗}(𝐱;{\text{H}}_{\text{1}})}{p_{𝐗}(𝐱;{\text{H}}_{\text{0}})}=\frac{\frac{\text{1}}{{\left(2\pi \right)}^{N/2}\text{\textbackslash{}hspace\{0.17em}}{\left|\Lambda \right|}^{1/2}}{exp}\left(-\frac{\text{1}}{\text{2}}(𝐱-𝐦{)}^{T}\Lambda^{-1}(𝐱-𝐦)\right)\}\frac{\text{1}}{\text{\textbackslash{}hspace\{0.17em}}(2\pi {)}^{N/2}exp\left(-\frac{\text{1}}{\text{2}}{𝐱}^{T}𝐱\right)\}>\gamma \text{\textbackslash{}hspace\{0.17em}\},\text{\textbackslash{}hspace\{0.17em}\;\;\;\;\;\;\;\;Decide\;\;H{\}}_{\text{1}}\text{\textbackslash{}hspace\{0.17em}\}. \end{array}$$ (24)

Similar to the case of univariate decision analysis (subsection **A**), after some simplifications, the test statistic *Z* can be obtained as follows:


$$\begin{array}{c} Z=\sum_{i=1}^{N}\{{X_{i}}^{\text{2}}-\lambda_{i}^{-1}(X_{i}-m_{i}{)}^{\text{2}}\}. \end{array}$$ (25)

and the binary hypothesis testing problem for the multivariate decision-making framework can be defined below:


$$\begin{array}{c} \begin{array}{c} {\text{H}}_{\text{0}}:X_{\text{1}}\;N\left(0,1\right),\text{\textbackslash{}hspace\{0.17em}\;...\;,\;\;and\}X_{N}\;N\left(0,1\right),\text{\textbackslash{}hspace\{0.17em}\;\}X_{\text{1}},\text{\textbackslash{}hspace\{0.17em}\;\}...\text{\textbackslash{}hspace\{0.17em}\},\text{\textbackslash{}hspace\{0.17em}\;and\;\;\}X_{N}\text{\textbackslash{}hspace\{0.17em}\;are\;\;independent,\}\\ {\text{H}}_{\text{1}}:X_{\text{1}}\;N\left(m_{\text{1}},\lambda_{\text{1}}\right),\text{...\textbackslash{}hspace\{0.17em}\},\text{\textbackslash{}hspace\{0.17em}\;and\}X_{N}\;N\left(m_{N},\lambda_{N}\right),\text{\textbackslash{}hspace\{0.17em}\;\;\}X_{\text{1}},\text{\textbackslash{}hspace\{0.17em}\;\}...\text{\textbackslash{}hspace\{0.17em}\},\text{\textbackslash{}hspace\{0.17em}\;\;and\;\;\}X_{N}\text{\textbackslash{}hspace\{0.17em}\;are\;\;independent.\} \end{array} \end{array}$$

The next step is finding the PDF of the test statistic *Z*, then computing the decision probabilities *P*_FA_ and *P*_D_. To do so, the characteristic function of the data under each hypothesis is needed. For the data under hypothesis H_0_, we have:


$$\begin{array}{c} \begin{array}{c} \varphi_{Z}(\omega \text{\textbackslash{}hspace\{0.17em}\};\text{\textbackslash{}hspace\{0.17em}H{\}}_{\text{0}})=E\left[exp\left(j\omega Z\right)\text{\textbackslash{}hspace\{0.17em}\};\text{\textbackslash{}hspace\{0.17em}H{\}}_{\text{0}}\right]=E\left[exp\left(j\omega \text{\textbackslash{}hspace\{0.17em}\}\sum_{i=1}^{N}\{{X_{i}}^{\text{2}}-\lambda_{i}^{-1}(X_{i}-m_{i}{)}^{\text{2}}\}\right)\text{\textbackslash{}hspace\{0.17em}\};\text{\textbackslash{}hspace\{0.17em}H{\}}_{\text{0}}\right]\\ =\text{\textbackslash{}hspace\{0.17em}\}\prod_{i=1}^{N}{\left(1-\frac{j2\omega \text{\textbackslash{}hspace\{0.17em}}{(}\lambda_{i}-1)\}\lambda_{i}\right)}^{-1/2}\text{\textbackslash{}hspace\{0.17em}exp\left({\frac{\omega \text{\textbackslash{}hspace\{0.17em}}{m}}_{i}^{\text{2}}(1-j2\omega )\}j\lambda_{i}+2\omega \text{\textbackslash{}hspace\{0.17em}(\lambda_{i}-1)\}\right)\} \end{array} \end{array}$$ (26)

and the characteristic function of the data hypothesis H_1_, will be calculated as below:


$$\begin{array}{c} \begin{array}{c} \varphi_{Z}(\omega \text{\textbackslash{}hspace\{0.17em}\};\text{\textbackslash{}hspace\{0.17em}H{\}}_{\text{1}})=E\left[exp\left(j\omega Z\right)\text{\textbackslash{}hspace\{0.17em}\};\text{\textbackslash{}hspace\{0.17em}H{\}}_{\text{1}}\right]=E\left[exp\left(j\omega \text{\textbackslash{}hspace\{0.17em}\}\sum_{i=1}^{N}\{{X_{i}}^{\text{2}}-\lambda_{i}^{-1}(X_{i}-m_{i}{)}^{\text{2}}\}\right)\text{\textbackslash{}hspace\{0.17em}\};\text{\textbackslash{}hspace\{0.17em}H{\}}_{\text{1}}\right]\\ =\text{\textbackslash{}hspace\{0.17em}\}\prod_{i=1}^{N}{\left(1-j2\omega \text{\textbackslash{}hspace\{0.17em}\}(\lambda_{i}-1)\right)}^{-1/2}\text{\textbackslash{}hspace\{0.17em}exp\left(-{\frac{\omega \text{\textbackslash{}hspace\{0.17em}}{m}}_{i}^{\text{2}}(1+j2\omega )\}j+2\omega \text{\textbackslash{}hspace\{0.17em}(\lambda_{i}-1)\}\right)\} \end{array} \end{array}$$ (27)

According to Equations (3)-(5), and similar to univariate decision analysis, *F*_*Z*_(*z*) can be numerically obtained using the characteristic function of *Z* in Equations (26) and (27), together with the Gil-Pelaez lemma. Eventually, we can find *P*_FA_ and *P*_D_.

Here, in the case of *N* = 2, we consider two time points, 30 minutes and 4 hours, to be simultaneously considered as the two desired variables for analyzing the decision-making framework using the experimental data set. The eigenvalue decompositions of all possible conditions analyzed here are provided in the SUPPLEMENTARY MATERIALS section.

Confidence intervals on *P*_D_ were obtained by non-parametric bootstrap: within each condition the single cells were resampled with replacement (1000 resamples), the lognormal parameters refit, and *P*_D_ recomputed; we report the 2.5–97.5 percentile interval.

Also, this approach can be developed to larger cellular signaling networks, including different in puts like secondary messengers or ligands, and the output could be various transcription factors [32].

### Optimal NP test for Gaussian-mixture hypotheses

In the mixed-population analysis (Figs 6 and 7), a fraction *β* of the cells are A20^−/−^ and the remaining 1 − *β* fraction are wild-type, so under each hypothesis the distribution of *y* = ln(nuclear NF-κB) is a two-component Gaussian mixture rather than a single Gaussian. With 𝒩(·; μ, σ²) being the Gaussian (normal) PDF introduced in Equation (7), the PDFs of the observations under the low- and high-TNF hypotheses are the two-component mixtures given in Equations (28) and (29), and the corresponding Neyman–Pearson-optimal test statistic is the log-likelihood ratio (LLR) of these two mixture PDFs, given in Equation (30):


$$\begin{array}{c} p(y;{\text{H}}_{\text{0}})=(1-\beta )N(y;\mu_{\text{W},\text{\textbackslash{}hspace\{0.17em}}0\},\sigma_{\text{W},\text{\textbackslash{}hspace\{0.17em}}0{\}}^{\text{2}})+\beta N(y;\mu_{\text{A},\text{\textbackslash{}hspace\{0.17em}}0\},\sigma_{\text{A},\text{\textbackslash{}hspace\{0.17em}}0{\}}^{\text{2}}) \end{array}$$ (28)

$$\begin{array}{c} p(y;{\text{H}}_{\text{1}})=(1-\beta )N(y;\mu_{\text{W},\text{\textbackslash{}hspace\{0.17em}}1\},\sigma_{\text{W},\text{\textbackslash{}hspace\{0.17em}}1{\}}^{\text{2}})+\beta N(y;\mu_{\text{A},\text{\textbackslash{}hspace\{0.17em}}1\},\sigma_{\text{A},\text{\textbackslash{}hspace\{0.17em}}1{\}}^{\text{2}}) \end{array}$$ (29)

$$\begin{array}{c} \ell_{\text{mix}}=ln(p;{\text{H}}_{\text{1}})-ln(p;{\text{H}}_{\text{0}}) \end{array}$$ (30)

In these equations, the subscripts W and A denote the wild-type and A20^−/−^ components, respectively, and the second subscript of 0 and 1 refer to the low and high TNF hypotheses, respectively. Unlike the single-Gaussian case this test statistic is not quadratic in the observation, so the characteristic-function (Gil-Pelaez) inversion used for the single-Gaussian case cannot be pursued in closed form here. We therefore set the threshold by Monte-Carlo: drawing 2 × 10^5^ samples from the H_0_ mixture, evaluating *ℓ*_mix_ for each, and taking the 1 − *P*_FA_ empirical quantile as the threshold γ. The detection probability is the fraction of an equally large H_1_ sample such that *ℓ*_mix_ ≥ γ_th_. Comparing this GM detector with the single-Gaussian detector used for Figs 6 and 7 (S7 Fig, S5 Table) gives agreement within |Δ*P*_D_| ≤ 0.017 at 30 minutes and ≤ 0.065 at 4 hours across all *β*, therefore, the single-Gaussian approximation closely tracks the optimal test while remaining analytically tractable.

## Supporting information

S1 Fig Receiver operating characteristic (ROC) curves for TNF–NF-κB signaling pathway evaluated using Neyman-Pearson theorem when low TNF H_0_ is 0.0021 ng/mL. A) Wild-type cells result for high TNF H_1_ equal to 0.2 ng/mL. B) A20^−/−^ cells results for high TNF H_1_ equal to 0.2 ng/mL. C) Wild-type cells result for high TNF H_1_ equal to 50 ng/mL. D) A20^−/−^ cells results for high TNF H_1_ equal to 50 ng/mL. E) Probability of Detection vs. various TNF levels for both wild-type and A20^−/−^ cells at different time points.
(TIF)

S2 Fig Receiver operating characteristic (ROC) curves for TNF–NF-κB signaling pathway evaluated using Neyman-Pearson theorem when low TNF H_0_ is 0.013 ng/mL. A) Wild-type cells result for high TNF H_1_ equal to 0.2 ng/mL. B) A20^−/−^ cells results for high TNF H_1_ equal to 0.2 ng/mL. C) Wild-type cells result for high TNF H_1_ equal to 50 ng/mL. D) A20^−/−^ cells results for high TNF H_1_ equal to 50 ng/mL. E) Probability of Detection vs. various TNF levels for both wild-type and A20^−/−^ cells at different time points.
(TIF)

S3 Fig Sensitivity of the detection behavior to the operating false alarm rate. ROC curves (*P*_D_ vs. *P*_FA_) for the four cell-types × time combinations at two representative decision problems (TNF 0.0052 vs. 0.2 ng/mL, left; TNF 0.0052 vs. 50 ng/mL, right). The dashed line marks the arbitrary reference operating point *P*_FA_ = 0.10. The observed ROC curve ordering — WT outperforming A20^−/−^ — is preserved across the entire range of *P*_FA_. Numerical *P*_D_ values at *P*_FA_ ∈ {0.01, 0.05, 0.10, 0.20, 0.50} are given in S1 Table.
(TIFF)

S4 Fig Maximum likelihood (equal-prior) versus Neyman–Pearson operating points. ROC curves for WT (blue) and A20^−/−^ (green) cells at 30 minutes, for TNF of 0.0052 vs. 0.2 ng/mL (left) and 0.0052 vs. 50 ng/mL (right). On each ROC curve, the triangle (▲) marks the equal-prior maximum likelihood operating point (decides H₁ when the likelihood ratio exceeds 1) and the circle (●) marks the Neyman–Pearson operating point at the fixed false alarm probability *P*_FA_ = 0.10 (dashed vertical line). Both the ML (▲) and NP (●) operating points lie on the same likelihood-ratio-based ROC curve and differ only in the chosen decision threshold (The slope of a line tangent to the ROC curve of a likelihood ratio detector at an operating point, *dP*_D_/ *dP*_FA_, is equal to the decision threshold of the detector at that point: at an ML operating point this slope is 1, that corresponds to the ROC curve tangent line that is parallel to the 45-degree reference line). The ML’s false alarm and detection probabilities together minimize the average decision error probability *P*_E_ = 0.5 (*P*_FA_ + (1 – *P*_D_)), whereas the NP’s *P*_D_ is maximized for each fixed *P*_FA_.
(TIFF)

S5 Fig Mixed-population detection under two decision models. Probability of detection *P*_D_ at fixed false alarm probability *P*_FA_ = 0.10 versus the A20^−/−^ fraction *β*, for the WT + A20^−/−^ mixture discriminating low (H₀ = 0.0052 ng/mL) from high (H₁ = 50 ng/mL) TNF. Purple: a single shared threshold applied by an external population observer (the Fig 6 detector). Green: a genotype-informed observer that gives each subpopulation its own optimal threshold (a common likelihood ratio threshold). (A) 30 min — the two models nearly coincide, the genotype-informed test marginally higher, because the mixture is near-Gaussian and the genotype label adds little. (B) 4 h — the single shared threshold is non-monotonic, falling to a minimum of ≈0.30 near *β* ≈ 0.3 because the elevated low-TNF (H₀) baseline of A20^−/−^ cells overlaps the high-TNF (H₁) responses of WT cells, which one threshold cannot separate; the genotype-informed observer is unaffected and stays well above. The genotype-informed test dominates at every *β* (the genotype label can only help) and reduces, when the label is known, to two ordinary single-population detections.
(TIFF)

S6 Fig Gaussian mixture response densities versus the single-Gaussian approximation in the mixed population. For a mixed population of a fraction *β* of A20^−/−^ cells and 1 − *β* wild-type cells, the distribution of ln(NF-κB) under each hypothesis is a two-component Gaussian mixture. Each panel overlays the exact mixture density (solid) on the moment-matched single Gaussian (dashed) for H_0_ (low TNF, 0.0052 ng/mL; blue) and H₁ (high TNF, 50 ng/mL; red), at mixing fractions *β* = 0.3, 0.5, 0.7 (columns) and at 30 minutes (top row) and 4 hours (bottom row). At 30 minutes the mixture is nearly Gaussian, and the single-Gaussian model tracks it closely; at 4 hours, both the H_0_ and H₁ mixture densities become visibly bimodal at intermediate *β* (most pronounced near *β* = 0.5), where the single-Gaussian approximation cannot mathematically capture two separate modes.
(TIFF)

S7 Fig Detection performance of the exact Gaussian mixture log-likelihood ratio test versus the single-Gaussian approximation test. Probability of detection *P*_D_ at fixed false alarm probability *P*_FA_ = 0.10 as a function of the A20^−/−^ fraction *β*, for the TNF 0.0052-versus-50 ng/mL decision scenario, at 30 minutes (left) and 4 hours (right). The exact Gaussian mixture log-likelihood ratio (GM-LLR) detector, for which the decision threshold is set by Monte-Carlo on the H₀ mixture (see METHODS) is compared with the single-Gaussian detector used in Figs 6 and 7. The two agree to within |Δ*P*_D_| ≤ 0.017 at 30 minutes and ≤ 0.065 at 4 hours across all *β*. Because the single-Gaussian *P*_D_ is evaluated using one Gaussian PDF fitted to a mixed Gaussian population, it is not the exact optimal *P*_D_ and may lie slightly above or below the GM-LLR *P*_D_.
(TIFF)

S8 Fig Goodness-of-fit of the lognormal model. Quantile–quantile (Q–Q) plots of ln(NF-κB) against the fitted normal distribution for 12 representative conditions (all four cell-types × time combinations at TNF = 0.0052, 0.2, and 50 ng/mL, spanning the entire TNF stimulation range). Each panel reports the sample size n and the Shapiro–Wilk (SW) p-value. Points close to the red identity line (reference line) indicate consistency with the lognormal model. While there are some visible departures in some panels, overall, the detection-related results and conclusions do not practically change much, as revealed upon using a model-free KDE-based detector (see S2B Table). Full goodness-of-fit statistics and results for all the 28 conditions appear in S2A Table.
(TIFF)

S1 Table Sensitivity of the detection results to the operating false alarm rate. *P*_D_ for each of the 48 H_0_-versus-H_1_ decision pairs at five operating points of *P*_FA_ ∈ {0.01, 0.05, 0.10, 0.20, 0.50}. The observed ordering of WT outperforming A20^−/−^ is preserved at every operating point. There are instances at the lowest dose (0.0021 ng/mL) and 4 h, where the WT/A20^−/−^ ordering is nearly the same or is reversed — a low-dose/late-time effect; it does not often happen and seems like an outlier compared to the general observed trend.
(DOCX)

S2 Table A. **Goodness-of-fit statistics for ln(NF-κB) across all 28 single-cell conditions (2 cell types × 2 time points × 7 doses).** Columns: sample size n, mean and standard deviation (SD) of ln(NF-κB), skewness, excess kurtosis, and Shapiro–Wilk p-value, Anderson–Darling A², and Jarque–Bera p-value. Shapiro–Wilk rejects strict normality at these large n, as expected [21]; the deviations are modest (|skewness| ≤ 1.0 in 22 of 28 conditions; |excess kurtosis| ≤ 3 in 25 of 28 conditions) [33]. **B. Robustness of the probability of detection to the lognormal assumption.** For each of the 48 H_0_-versus-H_1_ decision pairs (2 cell types × 2 time points × 3 low doses × 4 high doses), *P*_D_ at *P*_FA_ = 0.10 calculated for the lognormal likelihood ratio detector and a non-parametric KDE likelihood ratio detector, together with their absolute difference. Maximum |Δ*P*_D_| = 0.092; median |Δ*P*_D_| = 0.03.
(DOCX)

S3 Table Covariance diagnostics for the bivariate (30 minutes, 4 hours) analysis. For each cell type × TNF dose: cell counts at each time point, marginal standard deviations (SDs) of ln(NF-κB), the eigenvalues of the 2 × 2 covariance matrices, and their condition numbers. All condition numbers ≤ 1.63 and n ≥ 137, indicating well-conditioned covariance matrices.
(DOCX)

S4 Table Geometry of the Neyman–Pearson decision boundary for the 48 detection pairs. Since the log-likelihood ratio for Gaussian PDFs is quadratic, its decision region is generally specified by the two roots of the quadratic equation (Root 1, Root 2; in ln(NF-κB) units), listed together with the observed ln(NF-κB) data range. H_0_ and H_1_ denote the low- and high-TNF doses. If one of the roots falls outside the data range, we call it a secondary root, the H_0_ probability beyond the secondary root makes a negligible contribution to the false alarm probability (see the last column), and effectively we have a single threshold. However, if both roots fall inside the data range, we have two thresholds. Anyway, in every condition, the reported detection probability is computed using the exact decision region obtained by Monte Carlo, so no single-threshold approximation is made.
(DOCX)

S5 Table Single-Gaussian detector versus the exact Gaussian mixture log-likelihood ratio (GM-LLR) detector on the mixed population. *P*_D_ at *P*_FA_ = 0.10 for the TNF 0.0052-versus-50 ng/mL decision pair across the mixing fraction *β* ∈ {0,0.1,…,1.0}, at 30 minutes and 4 hours. Agreement is tight at 30 minutes (max |Δ*P*_D_| = 0.017) and within |Δ*P*_D_| ≤ 0.065 at 4 hours. Note: The single-Gaussian *P*_D_ is computed using one Gaussian PDF fitted to a mixed Gaussian population; therefore, its *P*_D_ values reported in Figs 6 and 7 are not exactly the same as the optimal GM-LLR *P*_D_ values. The *β* = 0 and *β* = 1 rows reproduce the pure-populations of WT and A20^−/−^ values of Table 2 within Monte-Carlo error (≤ 0.02). **Note:** The single-Gaussian *P*_D_ is computed using one Gaussian PDF fitted to a mixed Gaussian population; therefore, its *P*_D_ values reported in Figs 6 and 7 are not exactly the same as the optimal GM-LLR *P*_D_ values. The *β* = 0 and *β* = 1 rows reproduce the pure-populations of WT and A20^−/−^ values of Table 2 within Monte-Carlo error (≤ 0.02).
(DOCX)
