# Supplementary material for: Assessing the reliability of cellular decision making from noisy, multidimensional single-cell TNF–NF-κB signaling data
Source: PLoS Comput Biol. 2026 Aug 12;22(8):e1014608. doi: 10.1371/journal.pcbi.1014608 (PMC13552947; doi:10.1371/journal.pcbi.1014608)
Supplement: Supplementary file 1 [file pcbi.1014608.s009.docx]

**S1 Table. Sensitivity of the detection results to the operating false alarm rate.** *P*_D_ for each of the 48 H_0_-versus-H_1_ decision pairs at five operating points of *P*_FA_ ∈ {0.01, 0.05, 0.10, 0.20, 0.50}. The observed ordering of WT outperforming A20^-/-^ is preserved at every operating point. There are instances at the lowest dose (0.0021 ng/mL) and 4 h, where the WT/A20^-/-^ ordering is nearly the same or is reversed — a low-dose/late-time effect; it does not often happen and seems like an outlier compared to the general observed trend.

| **Cell-type × time** | **H_0_ TNF (ng/mL)** | **H_1_ TNF (ng/mL)** | **n(H_0_)** | **n(H_1_)** | ***P*_D_ (*P*_FA_=0.01)** | ***P*_D_ (*P*_FA_=0.05)** | ***P*_D_ (*P*_FA_=0.10)** | ***P*_D_ (*P*_FA_=0.20)** | ***P*_D_ (*P*_FA_=0.50)** |
| --- | --- | --- | --- | --- | --- | --- | --- | --- | --- |
| **WT 30 min** | 0.0021 | 0.2 | 378 | 671 | 0.285 | 0.533 | 0.665 | 0.801 | 0.950 |
| **WT 30 min** | 0.0021 | 0.51 | 378 | 344 | 0.657 | 0.848 | 0.912 | 0.960 | 0.994 |
| **WT 30 min** | 0.0021 | 1.3 | 378 | 355 | 0.720 | 0.885 | 0.936 | 0.972 | 0.996 |
| **WT 30 min** | 0.0021 | 50 | 378 | 442 | 0.900 | 0.976 | 0.991 | 0.997 | 1.000 |
| **WT 30 min** | 0.0052 | 0.2 | 377 | 671 | 0.351 | 0.583 | 0.699 | 0.816 | 0.948 |
| **WT 30 min** | 0.0052 | 0.51 | 377 | 344 | 0.720 | 0.874 | 0.926 | 0.964 | 0.994 |
| **WT 30 min** | 0.0052 | 1.3 | 377 | 355 | 0.776 | 0.906 | 0.946 | 0.975 | 0.996 |
| **WT 30 min** | 0.0052 | 50 | 377 | 442 | 0.931 | 0.983 | 0.993 | 0.998 | 1.000 |
| **WT 30 min** | 0.013 | 0.2 | 545 | 671 | 0.289 | 0.520 | 0.645 | 0.777 | 0.934 |
| **WT 30 min** | 0.013 | 0.51 | 545 | 344 | 0.661 | 0.840 | 0.904 | 0.953 | 0.991 |
| **WT 30 min** | 0.013 | 1.3 | 545 | 355 | 0.724 | 0.879 | 0.929 | 0.967 | 0.994 |
| **WT 30 min** | 0.013 | 50 | 545 | 442 | 0.902 | 0.975 | 0.989 | 0.997 | 1.000 |
| **A20^-/-^ 30 min** | 0.0021 | 0.2 | 367 | 348 | 0.024 | 0.119 | 0.230 | 0.421 | 0.798 |
| **A20^-/-^ 30 min** | 0.0021 | 0.51 | 367 | 313 | 0.081 | 0.267 | 0.415 | 0.610 | 0.891 |
| **A20^-/-^ 30 min** | 0.0021 | 1.3 | 367 | 355 | 0.160 | 0.420 | 0.586 | 0.767 | 0.956 |
| **A20^-/-^ 30 min** | 0.0021 | 50 | 367 | 311 | 0.193 | 0.477 | 0.645 | 0.815 | 0.970 |
| **A20^-/-^ 30 min** | 0.0052 | 0.2 | 343 | 348 | 0.029 | 0.127 | 0.232 | 0.404 | 0.758 |
| **A20^-/-^ 30 min** | 0.0052 | 0.51 | 343 | 313 | 0.100 | 0.282 | 0.418 | 0.596 | 0.866 |
| **A20^-/-^ 30 min** | 0.0052 | 1.3 | 343 | 355 | 0.191 | 0.439 | 0.590 | 0.755 | 0.942 |
| **A20^-/-^ 30 min** | 0.0052 | 50 | 343 | 311 | 0.228 | 0.496 | 0.649 | 0.804 | 0.960 |
| **A20^-/-^ 30 min** | 0.013 | 0.2 | 307 | 348 | 0.039 | 0.151 | 0.261 | 0.433 | 0.770 |
| **A20^-/-^ 30 min** | 0.013 | 0.51 | 307 | 313 | 0.123 | 0.315 | 0.450 | 0.621 | 0.873 |
| **A20^-/-^ 30 min** | 0.013 | 1.3 | 307 | 355 | 0.227 | 0.478 | 0.622 | 0.776 | 0.946 |
| **A20^-/-^ 30 min** | 0.013 | 50 | 307 | 311 | 0.269 | 0.537 | 0.681 | 0.823 | 0.964 |
| **WT 4 h** | 0.0021 | 0.2 | 419 | 401 | 0.038 | 0.151 | 0.262 | 0.436 | 0.775 |
| **WT 4 h** | 0.0021 | 0.51 | 419 | 476 | 0.063 | 0.226 | 0.366 | 0.563 | 0.867 |
| **WT 4 h** | 0.0021 | 1.3 | 419 | 396 | 0.110 | 0.300 | 0.438 | 0.614 | 0.875 |
| **WT 4 h** | 0.0021 | 50 | 419 | 601 | 0.120 | 0.332 | 0.482 | 0.665 | 0.909 |
| **WT 4 h** | 0.0052 | 0.2 | 542 | 401 | 0.094 | 0.270 | 0.403 | 0.580 | 0.856 |
| **WT 4 h** | 0.0052 | 0.51 | 542 | 476 | 0.147 | 0.375 | 0.528 | 0.705 | 0.924 |
| **WT 4 h** | 0.0052 | 1.3 | 542 | 396 | 0.214 | 0.446 | 0.584 | 0.737 | 0.925 |
| **WT 4 h** | 0.0052 | 50 | 542 | 601 | 0.237 | 0.490 | 0.634 | 0.785 | 0.949 |
| **WT 4 h** | 0.013 | 0.2 | 509 | 401 | 0.109 | 0.268 | 0.384 | 0.538 | 0.801 |
| **WT 4 h** | 0.013 | 0.51 | 509 | 476 | 0.169 | 0.374 | 0.506 | 0.664 | 0.887 |
| **WT 4 h** | 0.013 | 1.3 | 509 | 396 | 0.239 | 0.445 | 0.566 | 0.703 | 0.892 |
| **WT 4 h** | 0.013 | 50 | 509 | 601 | 0.265 | 0.489 | 0.615 | 0.752 | 0.922 |
| **A20^-/-^ 4 h** | 0.0021 | 0.2 | 318 | 233 | 0.039 | 0.149 | 0.255 | 0.421 | 0.754 |
| **A20^-/-^ 4 h** | 0.0021 | 0.51 | 318 | 474 | 0.031 | 0.125 | 0.220 | 0.377 | 0.715 |
| **A20^-/-^ 4 h** | 0.0021 | 1.3 | 318 | 209 | 0.124 | 0.314 | 0.447 | 0.615 | 0.867 |
| **A20^-/-^ 4 h** | 0.0021 | 50 | 318 | 137 | 0.199 | 0.472 | 0.634 | 0.801 | 0.964 |
| **A20^-/-^ 4 h** | 0.0052 | 0.2 | 192 | 233 | 0.014 | 0.069 | 0.135 | 0.263 | 0.602 |
| **A20^-/-^ 4 h** | 0.0052 | 0.51 | 192 | 474 | 0.012 | 0.057 | 0.115 | 0.230 | 0.558 |
| **A20^-/-^ 4 h** | 0.0052 | 1.3 | 192 | 209 | 0.058 | 0.184 | 0.294 | 0.456 | 0.765 |
| **A20^-/-^ 4 h** | 0.0052 | 50 | 192 | 137 | 0.090 | 0.291 | 0.447 | 0.645 | 0.910 |
| **A20^-/-^ 4 h** | 0.013 | 0.2 | 278 | 233 | 0.012 | 0.058 | 0.117 | 0.234 | 0.573 |
| **A20^-/-^ 4 h** | 0.013 | 0.51 | 278 | 474 | 0.012 | 0.058 | 0.115 | 0.229 | 0.563 |
| **A20^-/-^ 4 h** | 0.013 | 1.3 | 278 | 209 | 0.027 | 0.116 | 0.211 | 0.371 | 0.718 |
| **A20^-/-^ 4 h** | 0.013 | 50 | 278 | 137 | 0.041 | 0.187 | 0.332 | 0.546 | 0.879 |
