# Supplementary material for: Assessing the reliability of cellular decision making from noisy, multidimensional single-cell TNF–NF-κB signaling data
Source: PLoS Comput Biol. 2026 Aug 12;22(8):e1014608. doi: 10.1371/journal.pcbi.1014608 (PMC13552947; doi:10.1371/journal.pcbi.1014608)
Supplement: Supplementary file 2 [file pcbi.1014608.s010.docx]

**S2A Table.** **Goodness-of-fit statistics for ln(NF-κB) across all 28 single-cell conditions (2 cell types × 2 time points × 7 doses).**

Columns: sample size n, mean and standard deviation (SD) of ln(NF-κB), skewness, excess kurtosis, and Shapiro–Wilk p-value, Anderson–Darling A², and Jarque–Bera p-value. Shapiro–Wilk rejects strict normality at these large n, as expected [1]; the deviations are modest (|skewness| ≤ 1.0 in 22 of 28 conditions; |excess kurtosis| ≤ 3 in 25 of 28 conditions) [2].

| **Cell-type**  **×**  **time** | **TNF (ng/mL)** | **n** | **mean ln(NF-κB)** | **SD**  **ln(NF-κB)** | **skewness** | **excess kurtosis** | **Shapiro–Wilk p-value** | **Anderson–Darling A²** | **Jarque–Bera p-value** |
| --- | --- | --- | --- | --- | --- | --- | --- | --- | --- |
| **WT 30 min** | 0.0021 | 378 | 8.085 | 0.190 | 0.58 | 0.21 | 7.9×10⁻⁶ | 2.439 | 1.5×10⁻⁵ |
| **WT 30 min** | 0.0052 | 377 | 8.088 | 0.172 | 0.68 | 0.80 | 1.9×10⁻⁶ | 2.065 | 3.0×10⁻⁹ |
| **WT 30 min** | 0.013 | 545 | 8.112 | 0.177 | 0.57 | 0.95 | 2.4×10⁻⁷ | 2.700 | 1.4×10⁻¹¹ |
| **WT 30 min** | 0.2 | 671 | 8.413 | 0.200 | 0.18 | 0.05 | 0.257 | 0.316 | 0.166 |
| **WT 30 min** | 0.51 | 344 | 8.611 | 0.209 | 0.06 | -0.15 | 0.874 | 0.170 | 0.775 |
| **WT 30 min** | 1.3 | 355 | 8.649 | 0.211 | -0.16 | 0.15 | 0.680 | 0.397 | 0.378 |
| **WT 30 min** | 50 | 442 | 8.763 | 0.184 | 0.36 | 0.41 | 0.013 | 0.890 | 0.002 |
| **A20^-/-^ 30 min** | 0.0021 | 367 | 8.292 | 0.245 | 0.99 | 2.13 | 2.2×10⁻⁹ | 3.301 | 7.6×10⁻²⁹ |
| **A20^-/-^ 30 min** | 0.0052 | 343 | 8.319 | 0.223 | 1.14 | 2.52 | 8.3×10⁻¹¹ | 4.113 | 1.4×10⁻³⁶ |
| **A20^-/-^ 30 min** | 0.013 | 307 | 8.312 | 0.215 | 1.22 | 2.90 | 9.0×10⁻¹¹ | 4.856 | 1.3×10⁻⁴⁰ |
| **A20^-/-^ 30 min** | 0.2 | 348 | 8.458 | 0.200 | 0.31 | 1.24 | 0.002 | 1.078 | 8.0×10⁻⁷ |
| **A20^-/-^ 30 min** | 0.51 | 313 | 8.560 | 0.218 | 0.79 | 1.87 | 1.6×10⁻⁶ | 1.478 | 1.3×10⁻¹⁷ |
| **A20^-/-^ 30 min** | 1.3 | 355 | 8.653 | 0.212 | 0.74 | 2.17 | 7.5×10⁻⁷ | 1.666 | 6.9×10⁻²³ |
| **A20^-/-^ 30 min** | 50 | 311 | 8.684 | 0.208 | 0.00 | 0.14 | 0.794 | 0.225 | 0.886 |
| **WT 4 h** | 0.0021 | 419 | 8.043 | 0.220 | 1.91 | 6.91 | 3.2×10⁻¹⁸ | 10.079 | 2.6×10⁻²³⁷ |
| **WT 4 h** | 0.0052 | 542 | 7.981 | 0.207 | 2.37 | 10.70 | 3.1×10⁻²³ | 12.686 | <10⁻³⁰⁰ |
| **WT 4 h** | 0.013 | 509 | 8.025 | 0.180 | 1.63 | 10.33 | 8.6×10⁻¹⁶ | 2.927 | <10⁻³⁰⁰ |
| **WT 4 h** | 0.2 | 401 | 8.195 | 0.203 | 0.35 | -0.09 | 0.003 | 1.048 | 0.016 |
| **WT 4 h** | 0.51 | 476 | 8.259 | 0.194 | 0.26 | -0.21 | 0.034 | 0.826 | 0.048 |
| **WT 4 h** | 1.3 | 396 | 8.291 | 0.216 | 0.44 | 0.12 | 9.0×10⁻⁴ | 1.471 | 0.002 |
| **WT 4 h** | 50 | 601 | 8.316 | 0.205 | 0.45 | 0.51 | 7.1×10⁻⁵ | 1.688 | 1.4×10⁻⁶ |
| **A20^-/-^ 4 h** | 0.0021 | 318 | 8.441 | 0.248 | 0.72 | 0.81 | 2.5×10⁻⁶ | 1.812 | 1.2×10⁻⁸ |
| **A20^-/-^ 4 h** | 0.0052 | 192 | 8.542 | 0.251 | 0.66 | 1.88 | 0.001 | 0.603 | 6.8×10⁻¹⁰ |
| **A20^-/-^ 4 h** | 0.013 | 278 | 8.579 | 0.274 | 1.09 | 1.70 | 4.1×10⁻¹⁰ | 5.357 | 5.8×10⁻²⁰ |
| **A20^-/-^ 4 h** | 0.2 | 233 | 8.603 | 0.237 | 0.66 | 0.70 | 1.5×10⁻⁴ | 1.129 | 2.1×10⁻⁵ |
| **A20^-/-^ 4 h** | 0.51 | 474 | 8.575 | 0.238 | 0.87 | 2.30 | 3.0×10⁻¹⁰ | 4.276 | 2.1×10⁻³⁶ |
| **A20^-/-^ 4 h** | 1.3 | 209 | 8.725 | 0.255 | 0.82 | 1.66 | 3.1×10⁻⁵ | 1.378 | 5.8×10⁻¹¹ |
| **A20^-/-^ 4 h** | 50 | 137 | 8.834 | 0.219 | 0.20 | -0.14 | 0.662 | 0.279 | 0.607 |

**References**

1. Ghasemi A, Zahediasl S. Normality tests for statistical analysis: a guide for non-statisticians. Int J Endocrinol Metab. 2012;10(2):486–9.
2. D'Agostino RB, Stephens MA, editors. Goodness-of-Fit Techniques. New York: Marcel Dekker; 1986.

**S2B Table.** **Robustness of the probability of detection to the lognormal assumption.**

For each of the 48 H_0_-versus-H_1_ decision pairs (2 cell types × 2 time points × 3 low doses × 4 high doses), *P*_D_ at *P*_FA_ = 0.10 calculated for the lognormal likelihood ratio detector and a non-parametric KDE likelihood ratio detector, together with their absolute difference. Maximum |Δ*P*_D_| = 0.092; median |Δ*P*_D_| = 0.03.

| **Cell-type × time** | **H_0_ TNF (ng/mL)** | **H_1_ TNF (ng/mL)** | ***P*_D_ (lognormal)** | ***P*_D_ (KDE)** | **\|Δ*P*_D_\|** |
| --- | --- | --- | --- | --- | --- |
| **WT 30 min** | 0.0021 | 0.2 | 0.665 | 0.599 | 0.066 |
| **WT 30 min** | 0.0021 | 0.51 | 0.912 | 0.870 | 0.042 |
| **WT 30 min** | 0.0021 | 1.3 | 0.936 | 0.902 | 0.034 |
| **WT 30 min** | 0.0021 | 50 | 0.991 | 0.989 | 0.002 |
| **WT 30 min** | 0.0052 | 0.2 | 0.699 | 0.652 | 0.047 |
| **WT 30 min** | 0.0052 | 0.51 | 0.926 | 0.896 | 0.029 |
| **WT 30 min** | 0.0052 | 1.3 | 0.946 | 0.922 | 0.024 |
| **WT 30 min** | 0.0052 | 50 | 0.993 | 0.992 | 0.000 |
| **WT 30 min** | 0.013 | 0.2 | 0.645 | 0.608 | 0.037 |
| **WT 30 min** | 0.013 | 0.51 | 0.904 | 0.874 | 0.029 |
| **WT 30 min** | 0.013 | 1.3 | 0.929 | 0.906 | 0.024 |
| **WT 30 min** | 0.013 | 50 | 0.989 | 0.989 | 0.000 |
| **A20^-/-^ 30 min** | 0.0021 | 0.2 | 0.230 | 0.243 | 0.013 |
| **A20^-/-^ 30 min** | 0.0021 | 0.51 | 0.415 | 0.389 | 0.026 |
| **A20^-/-^ 30 min** | 0.0021 | 1.3 | 0.586 | 0.540 | 0.047 |
| **A20^-/-^ 30 min** | 0.0021 | 50 | 0.645 | 0.626 | 0.020 |
| **A20^-/-^ 30 min** | 0.0052 | 0.2 | 0.232 | 0.259 | 0.027 |
| **A20^-/-^ 30 min** | 0.0052 | 0.51 | 0.418 | 0.420 | 0.001 |
| **A20^-/-^ 30 min** | 0.0052 | 1.3 | 0.590 | 0.591 | 0.001 |
| **A20^-/-^ 30 min** | 0.0052 | 50 | 0.649 | 0.662 | 0.014 |
| **A20^-/-^ 30 min** | 0.013 | 0.2 | 0.261 | 0.233 | 0.028 |
| **A20^-/-^ 30 min** | 0.013 | 0.51 | 0.450 | 0.392 | 0.059 |
| **A20^-/-^ 30 min** | 0.013 | 1.3 | 0.622 | 0.568 | 0.054 |
| **A20^-/-^ 30 min** | 0.013 | 50 | 0.681 | 0.648 | 0.032 |
| **WT 4 h** | 0.0021 | 0.2 | 0.262 | 0.323 | 0.061 |
| **WT 4 h** | 0.0021 | 0.51 | 0.366 | 0.411 | 0.045 |
| **WT 4 h** | 0.0021 | 1.3 | 0.438 | 0.453 | 0.015 |
| **WT 4 h** | 0.0021 | 50 | 0.482 | 0.500 | 0.018 |
| **WT 4 h** | 0.0052 | 0.2 | 0.403 | 0.495 | 0.092 |
| **WT 4 h** | 0.0052 | 0.51 | 0.528 | 0.619 | 0.092 |
| **WT 4 h** | 0.0052 | 1.3 | 0.584 | 0.638 | 0.054 |
| **WT 4 h** | 0.0052 | 50 | 0.634 | 0.717 | 0.083 |
| **WT 4 h** | 0.013 | 0.2 | 0.384 | 0.391 | 0.008 |
| **WT 4 h** | 0.013 | 0.51 | 0.506 | 0.494 | 0.012 |
| **WT 4 h** | 0.013 | 1.3 | 0.566 | 0.535 | 0.031 |
| **WT 4 h** | 0.013 | 50 | 0.615 | 0.605 | 0.010 |
| **A20^-/-^ 4 h** | 0.0021 | 0.2 | 0.255 | 0.231 | 0.024 |
| **A20^-/-^ 4 h** | 0.0021 | 0.51 | 0.220 | 0.182 | 0.038 |
| **A20^-/-^ 4 h** | 0.0021 | 1.3 | 0.447 | 0.402 | 0.045 |
| **A20^-/-^ 4 h** | 0.0021 | 50 | 0.634 | 0.602 | 0.033 |
| **A20^-/-^ 4 h** | 0.0052 | 0.2 | 0.135 | 0.141 | 0.006 |
| **A20^-/-^ 4 h** | 0.0052 | 0.51 | 0.115 | 0.135 | 0.019 |
| **A20^-/-^ 4 h** | 0.0052 | 1.3 | 0.294 | 0.260 | 0.035 |
| **A20^-/-^ 4 h** | 0.0052 | 50 | 0.447 | 0.454 | 0.008 |
| **A20^-/-^ 4 h** | 0.013 | 0.2 | 0.117 | 0.145 | 0.028 |
| **A20^-/-^ 4 h** | 0.013 | 0.51 | 0.115 | 0.126 | 0.011 |
| **A20^-/-^ 4 h** | 0.013 | 1.3 | 0.211 | 0.269 | 0.058 |
| **A20^-/-^ 4 h** | 0.013 | 50 | 0.332 | 0.424 | 0.092 |
