# Supplementary material for: Assessing the reliability of cellular decision making from noisy, multidimensional single-cell TNF–NF-κB signaling data
Source: PLoS Comput Biol. 2026 Aug 12;22(8):e1014608. doi: 10.1371/journal.pcbi.1014608 (PMC13552947; doi:10.1371/journal.pcbi.1014608)
Supplement: Supplementary file 3 [file pcbi.1014608.s011.docx]

**S3 Table. Covariance diagnostics for the bivariate (30 minutes, 4 hours) analysis.** For each cell type × TNF dose: cell counts at each time point, marginal standard deviations (SDs) of ln(NF-κB), the eigenvalues of the 2×2 covariance matrices, and their condition numbers. All condition numbers ≤ 1.63 and n ≥ 137, indicating well-conditioned covariance matrices.

| **Cell type** | **TNF (ng/mL)** | **n**  **(30 minutes)** | **n**  **(4 hours)** | **SD  (30 minutes)** | **SD**  **(4 hours)** | **Covariance matrix eigenvalue min** | **Covariance matrix eigenvalue max** | **Covariance matrix condition number** |
| --- | --- | --- | --- | --- | --- | --- | --- | --- |
| **WT** | **0.0021** | 378 | 419 | 0.190 | 0.220 | 0.0359 | 0.0483 | 1.347 |
| **WT** | **0.0052** | 377 | 542 | 0.172 | 0.207 | 0.0296 | 0.0427 | 1.444 |
| **WT** | **0.013** | 545 | 509 | 0.177 | 0.180 | 0.0313 | 0.0325 | 1.038 |
| **WT** | **0.2** | 671 | 401 | 0.200 | 0.203 | 0.0399 | 0.0411 | 1.031 |
| **WT** | **0.51** | 344 | 476 | 0.209 | 0.194 | 0.0376 | 0.0437 | 1.162 |
| **WT** | **1.3** | 355 | 396 | 0.211 | 0.216 | 0.0446 | 0.0467 | 1.047 |
| **WT** | **50** | 442 | 601 | 0.184 | 0.205 | 0.0339 | 0.0420 | 1.237 |
| **A20^-/-^** | **0.0021** | 367 | 318 | 0.245 | 0.248 | 0.0603 | 0.0615 | 1.021 |
| **A20^-/-^** | **0.0052** | 343 | 192 | 0.223 | 0.251 | 0.0496 | 0.0630 | 1.268 |
| **A20^-/-^** | **0.013** | 307 | 278 | 0.215 | 0.274 | 0.0461 | 0.0750 | 1.626 |
| **A20^-/-^** | **0.2** | 348 | 233 | 0.200 | 0.237 | 0.0401 | 0.0560 | 1.397 |
| **A20^-/-^** | **0.51** | 313 | 474 | 0.218 | 0.238 | 0.0475 | 0.0566 | 1.192 |
| **A20^-/-^** | **1.3** | 355 | 209 | 0.212 | 0.255 | 0.0450 | 0.0652 | 1.448 |
| **A20^-/-^** | **50** | 311 | 137 | 0.208 | 0.219 | 0.0431 | 0.0478 | 1.111 |
