# Supplementary material for: Assessing the reliability of cellular decision making from noisy, multidimensional single-cell TNF–NF-κB signaling data
Source: PLoS Comput Biol. 2026 Aug 12;22(8):e1014608. doi: 10.1371/journal.pcbi.1014608 (PMC13552947; doi:10.1371/journal.pcbi.1014608)
Supplement: Supplementary file 4 [file pcbi.1014608.s012.docx]

**S4 Table. Geometry of the Neyman–Pearson decision boundary for the 48 detection pairs.** Since the log-likelihood ratio for Gaussian PDFs is quadratic, its decision region is generally specified by the two roots of the quadratic equation (Root 1, Root 2; in ln(NF-κB) units), listed together with the observed ln(NF-κB) data range. H_0_ and H_1_ denote the low- and high-TNF doses. If one of the roots falls outside the data range, we call it a secondary root, the H_0_ probability beyond the secondary root makes a negligible contribution to the false alarm probability (see the last column), and effectively we have a single threshold. However, if both roots fall inside the data range, we have two thresholds. Anyway, in every condition, the reported detection probability is computed using the exact decision region obtained by Monte Carlo, so no single-threshold approximation is made.

| **Cell-type × time** | **H_0_ (ng/mL)** | **H_1_**  **(ng/mL)** | **Root 1** | **Root 2** | **Data range** | **Both roots within the data range** | **H_0_ probability beyond the secondary root** |
| --- | --- | --- | --- | --- | --- | --- | --- |
| **WT 30 min** | 0.0021 | 0.2 | 1.989 | 8.328 | 7.62–9.11 | no | < 1e-10 |
| **WT 30 min** | 0.0021 | 0.51 | 3.006 | 8.328 | 7.62–9.29 | no | < 1e-10 |
| **WT 30 min** | 0.0021 | 1.3 | 3.178 | 8.327 | 7.62–9.26 | no | < 1e-10 |
| **WT 30 min** | 0.0021 | 50.0 | 8.327 | 32.474 | 7.62–9.48 | no | < 1e-10 |
| **WT 30 min** | 0.0052 | 0.2 | 6.02 | 8.309 | 7.66–9.11 | no | < 1e-10 |
| **WT 30 min** | 0.0052 | 0.51 | 5.688 | 8.309 | 7.66–9.29 | no | < 1e-10 |
| **WT 30 min** | 0.0052 | 1.3 | 5.664 | 8.309 | 7.66–9.26 | no | < 1e-10 |
| **WT 30 min** | 0.0052 | 50.0 | -1.249 | 8.309 | 7.66–9.48 | no | < 1e-10 |
| **WT 30 min** | 0.013 | 0.2 | 5.71 | 8.339 | 7.44–9.11 | no | < 1e-10 |
| **WT 30 min** | 0.013 | 0.51 | 5.377 | 8.339 | 7.44–9.29 | no | < 1e-10 |
| **WT 30 min** | 0.013 | 1.3 | 5.362 | 8.339 | 7.44–9.26 | no | < 1e-10 |
| **WT 30 min** | 0.013 | 50.0 | -7.474 | 8.339 | 7.44–9.48 | no | < 1e-10 |
| **A20^-/-^ 30 min** | 0.0021 | 0.2 | 8.603 | 8.975 | 7.75–9.57 | yes | NA |
| **A20^-/-^ 30 min** | 0.0021 | 0.51 | 8.606 | 10.508 | 7.75–9.57 | no | < 1e-10 |
| **A20^-/-^ 30 min** | 0.0021 | 1.3 | 8.606 | 10.835 | 7.75–9.67 | no | < 1e-10 |
| **A20^-/-^ 30 min** | 0.0021 | 50.0 | 8.607 | 10.727 | 7.75–9.57 | no | < 1e-10 |
| **A20^-/-^ 30 min** | 0.0052 | 0.2 | 8.605 | 9.479 | 7.83–9.31 | no | 9.7e-08 |
| **A20^-/-^ 30 min** | 0.0052 | 0.51 | 8.605 | 19.211 | 7.85–9.57 | no | < 1e-10 |
| **A20^-/-^ 30 min** | 0.0052 | 1.3 | 8.605 | 15.22 | 7.85–9.67 | no | < 1e-10 |
| **A20^-/-^ 30 min** | 0.0052 | 50.0 | 8.605 | 13.55 | 7.85–9.33 | no | < 1e-10 |
| **A20^-/-^ 30 min** | 0.013 | 0.2 | 8.586 | 10.277 | 7.83–9.47 | no | < 1e-10 |
| **A20^-/-^ 30 min** | 0.013 | 0.51 | -8.654 | 8.587 | 7.91–9.57 | no | < 1e-10 |
| **A20^-/-^ 30 min** | 0.013 | 1.3 | 8.587 | 36.625 | 7.91–9.67 | no | < 1e-10 |
| **A20^-/-^ 30 min** | 0.013 | 50.0 | 8.587 | 19.279 | 7.91–9.47 | no | < 1e-10 |
| **WT 4 h** | 0.0021 | 0.2 | 8.325 | 9.81 | 7.58–9.3 | no | < 1e-10 |
| **WT 4 h** | 0.0021 | 0.51 | 8.325 | 9.705 | 7.58–9.3 | no | < 1e-10 |
| **WT 4 h** | 0.0021 | 1.3 | 8.325 | 22.366 | 7.58–9.3 | no | < 1e-10 |
| **WT 4 h** | 0.0021 | 50.0 | 8.325 | 11.9 | 7.58–9.3 | no | < 1e-10 |
| **WT 4 h** | 0.0052 | 0.2 | 8.246 | 19.775 | 7.62–9.34 | no | < 1e-10 |
| **WT 4 h** | 0.0052 | 0.51 | 8.246 | 12.416 | 7.62–9.34 | no | < 1e-10 |
| **WT 4 h** | 0.0052 | 1.3 | 1.135 | 8.245 | 7.62–9.34 | no | < 1e-10 |
| **WT 4 h** | 0.0052 | 50.0 | 8.246 | 48.831 | 7.62–9.34 | no | < 1e-10 |
| **WT 4 h** | 0.013 | 0.2 | 6.516 | 8.256 | 7.65–9.58 | no | < 1e-10 |
| **WT 4 h** | 0.013 | 0.51 | 4.846 | 8.256 | 7.65–9.58 | no | < 1e-10 |
| **WT 4 h** | 0.013 | 1.3 | 6.578 | 8.256 | 7.65–9.58 | no | < 1e-10 |
| **WT 4 h** | 0.013 | 50.0 | 5.807 | 8.256 | 7.65–9.58 | no | < 1e-10 |
| **A20^-/-^ 4 h** | 0.0021 | 0.2 | 8.759 | 11.738 | 7.95–9.54 | no | < 1e-10 |
| **A20^-/-^ 4 h** | 0.0021 | 0.51 | 8.759 | 11.516 | 7.95–9.75 | no | < 1e-10 |
| **A20^-/-^ 4 h** | 0.0021 | 1.3 | -1.35 | 8.759 | 7.95–9.78 | no | < 1e-10 |
| **A20^-/-^ 4 h** | 0.0021 | 50.0 | 8.759 | 11.658 | 7.95–9.41 | no | < 1e-10 |
| **A20^-/-^ 4 h** | 0.0052 | 0.2 | 8.862 | 9.328 | 7.92–9.69 | yes | NA |
| **A20^-/-^ 4 h** | 0.0052 | 0.51 | 8.801 | 8.953 | 7.92–9.75 | yes | NA |
| **A20^-/-^ 4 h** | 0.0052 | 1.3 | -2.061 | 8.863 | 7.92–9.78 | no | < 1e-10 |
| **A20^-/-^ 4 h** | 0.0052 | 50.0 | 8.863 | 10.655 | 7.92–9.69 | no | < 1e-10 |
| **A20^-/-^ 4 h** | 0.013 | 0.2 | 8.637 | 8.711 | 8.0–9.59 | yes | NA |
| **A20^-/-^ 4 h** | 0.013 | 0.51 | 8.531 | 8.6 | 7.99–9.75 | yes | NA |
| **A20^-/-^ 4 h** | 0.013 | 1.3 | 8.929 | 10.463 | 8.0–9.78 | no | < 1e-10 |
| **A20^-/-^ 4 h** | 0.013 | 50.0 | 8.93 | 9.638 | 8.0–9.59 | no | 5.5e-05 |
