# Supplementary material for: Assessing the reliability of cellular decision making from noisy, multidimensional single-cell TNF–NF-κB signaling data
Source: PLoS Comput Biol. 2026 Aug 12;22(8):e1014608. doi: 10.1371/journal.pcbi.1014608 (PMC13552947; doi:10.1371/journal.pcbi.1014608)
Supplement: Supplementary file 5 [file pcbi.1014608.s013.docx]

**S5 Table. Single-Gaussian detector versus the exact Gaussian mixture log-likelihood ratio (GM-LLR) detector on the mixed population.** *P*_D_ at *P*_FA_ = 0.10 for the TNF 0.0052-versus-50 ng/mL decision pair across the mixing fraction β ∈ {0,0.1,…,1.0}, at 30 minutes and 4 hours. Agreement is tight at 30 minutes (max |Δ*P*_D_| = 0.017) and within |Δ*P*_D_| ≤ 0.065 at 4 hours.

| **Time** | ***β*** | ***P*_D_ (single-Gaussian fit)** | ***P*_D_ (GMM-LLR)** | **\|Δ*P*_D_\|** |
| --- | --- | --- | --- | --- |
| **30 min** | 0.0 | 0.993 | 0.993 | 0.000 |
| **30 min** | 0.1 | 0.983 | 0.983 | 0.000 |
| **30 min** | 0.2 | 0.966 | 0.963 | 0.003 |
| **30 min** | 0.3 | 0.941 | 0.934 | 0.006 |
| **30 min** | 0.4 | 0.908 | 0.896 | 0.012 |
| **30 min** | 0.5 | 0.870 | 0.854 | 0.016 |
| **30 min** | 0.6 | 0.826 | 0.809 | 0.017 |
| **30 min** | 0.7 | 0.781 | 0.769 | 0.011 |
| **30 min** | 0.8 | 0.735 | 0.727 | 0.007 |
| **30 min** | 0.9 | 0.690 | 0.689 | 0.001 |
| **30 min** | 1.0 | 0.649 | 0.650 | 0.002 |
| **4 h** | 0.0 | 0.634 | 0.633 | 0.001 |
| **4 h** | 0.1 | 0.476 | 0.456 | 0.020 |
| **4 h** | 0.2 | 0.402 | 0.340 | 0.062 |
| **4 h** | 0.3 | 0.362 | 0.297 | 0.064 |
| **4 h** | 0.4 | 0.338 | 0.302 | 0.037 |
| **4 h** | 0.5 | 0.326 | 0.329 | 0.003 |
| **4 h** | 0.6 | 0.322 | 0.356 | 0.034 |
| **4 h** | 0.7 | 0.326 | 0.383 | 0.057 |
| **4 h** | 0.8 | 0.341 | 0.406 | 0.065 |
| **4 h** | 0.9 | 0.374 | 0.430 | 0.056 |
| **4 h** | 1.0 | 0.447 | 0.448 | 0.002 |

**Note:** The single-Gaussian *P*_D_ is computed using one Gaussian PDF fitted to a mixed Gaussian population; therefore, its *P*_D_ values reported in Figs 6 and 7 are not exactly the same as the optimal GM-LLR *P*_D_ values. The *β* = 0 and *β* = 1 rows reproduce the pure-populations of WT and A20^-/-^ values of Table 2 within Monte-Carlo error (≤ 0.02).
